# Supplementary material for: The Antifungal Action Mode of N-Phenacyldibromobenzimidazoles
Source: Molecules. 2021 Sep 8;26(18):5463. doi: 10.3390/molecules26185463 (PMC8465355; doi:10.3390/molecules26185463)
Supplement: Supplementary file 1 [file molecules-26-05463-s001.zip › molecules-1345012-supplementary.pdf]

Supplementary Materials

The Antifungal Action Mode of N-phenacyldibromobenzimidazoles

Monika Staniszewska[1,*], Łukasz Kuryk[2,3], Aleksander Gryciuk[4], Joanna Kawalec[4], Marta Rogalska[4], Joanna Baran[1,4], Anna Kowalkowska[4,]**

[1,*]Warsaw University of Technology, Centre for Advanced Materials and Technologies CEZAMAT, Poleczki 19, 02-822, Poland, mstaniszewska@pw.edu.pl (MS)

[2] Department of Virology, National Institute of Public Health-National Institute of Hygiene, Chocimska 24, 00-791 Warsaw, Poland

[3]Clinical Science, Targovax Oy, Saukonpaadenranta 2, 00180 Helsinki, Finland

[4]*Warsaw University of Technology, Faculty of Chemistry, Noakowskiego St 3, 00-664 Warsaw, Poland, e-mail: anna.kowalkowska@pw.edu.pl (AK)

Table of Contents

1. Analytical data of compounds 4a, 4j, 5b, 5d, 5e, 5f, 5h, 5j — S2

2. ^{1}H and ^{13}C NMR of compounds 4a, 4j, 5b, 5d, 5e, 5f, 5h, 5j — S4

3. HRMS of compounds 4a, 4j, 5b, 5d, 5e, 5f, 5h, 5j — S21

4. Antifungal assays — S29

1. Analytical data of compounds 4a, 4j, 5b, 5d, 5e, 5f, 5h, 5j

2-(5,6-dibromo-1*H*-benzimidazol-1-yl)-1-phenylethanone (**4a**): white crystals, m.p. = 197-198 °C (EtOH); ^{1}H NMR (500 MHz, DMSO-d_6) δ = δ = 6.06 (s, 2H, CH_2), 7.62-7.65 (m, 2H, C_6H_5), 7.73-7.77 (m, 1H, C_6H_5), 8.08-8.10 (m, 2H, C_6H_5), 8.11 (s, 1H, C_6H_2), 8.20 (s, 1H, C_6H_2), 8.27 (s, 1H, N=CH); ^{13}C NMR (125 MHz, DMSO-d_6) δ = 51.21, 115.84, 116.72, 123.63, 128.18, 128.86, 134.02, 134.33, 135.24, 143.62, 147.30, 192.93; HRMS: calculated for $C_{15}H_{11}Br_2N_2O$ $[M+H]^+$: 394.92122. Found: 394.92130.

2-(5,6-dibromo-1*H*-benzimidazol-1-yl)-1-(2,4,6-trifluorophenyl)ethanone (**4j**): white crystals, m.p. 137-139 °C; ^{1}H NMR (500 MHz, DMSO-d_6) δ = 5.77 (s, 2H, CH_2), 7.47 (td, J = 9.29 Hz, J = 2.45 Hz, 2H, $C_6H_2F_3$), 8.10 (s, 1H, C_6H_2), 8.14 (s, 1H, C_6H_2), 8.27 (d, J = 1.47 Hz, 1H, N=CH); ^{13}C NMR (125 MHz, DMSO-d_6) δ = 54.65-54.75 (m), 101.84-102.30 (m), 115.77, 115.99, 116.87, 123.66, 134.91, 143.55, 147.21, 161.71 (ddd, J = 257.26 Hz, J = 16.63 Hz, J = 9.78 Hz), 163.32-165.60 (m), 188.59-188.64 (m); HRMS: calculated for $C_{15}H_8Br_2F_3N_2O$ $[M+H]^+$: 448.89295. Found: 448.89326.

2-(4,6-dibromo-1*H*-benzimidazol-1-yl)-1-(4-fluorophenyl)ethanone (**5b**): white crystals, m.p. 177-178 °C; ^{1}H NMR (500 MHz, DMSO-d_6) δ = δ = 6.05 (s, 2H, CH_2), 7.45-7.50 (m, 2H, C_6H_4), 7.64 (d, J = 1.47 Hz, 1H, C_6H_2), 8.02 (d, J = 1.47 Hz, 1H, C_6H_2), 8.15-8.19 (m, 2H, C_6H_4), 8.30 (s, 1H, N=CH); ^{13}C NMR (125 MHz, DMSO-d_6) δ = 51.38, 113.21, 113.74, 114.87, 115.97 (d, J = 22.50 Hz), 126.50, 131.11 (d, J = 2.94 Hz), 131.28 (d, J = 9.78 Hz), 136.27, 140.84, 146.55, 165.46 (d, J = 252.37 Hz), 191.53; HRMS: calculated for $C_{15}H_{10}Br_2FN_2O$ $[M+H]^+$: 412.91180. Found: 412.91185.

2-(4,6-dibromo-1*H*-benzimidazol-1-yl)-1-(4-bromophenyl)ethanone (**5d**): white crystals, m.p. 196-197 °C; ^{1}H NMR (500 MHz, DMSO-d_6) δ = 6.04 (s, 2H, CH_2), 7.64 (d, J = 1.96 Hz, 1H, C_6H_2), 7.85-7.87 (m, 2H, C_6H_4), 8.00-8.03 (m, 2H from C_6H_4 and 1H from C_6H_2), 8.30 (s, 1H, N=CH); ^{13}C NMR (125 MHz, DMSO-d_6) δ = 51.43, 113.20, 113.76, 114.87, 126.50, 128.12, 130.15, 131.94, 133.34, 136.24, 140.83, 146.51, 192.21; HRMS: calculated for $C_{15}H_{10}Br_3N_2O$ $[M+H]^+$: 472.83173. Found: 472.83233.

2-(4,6-dibromo-1*H*-benzimidazol-1-yl)-1-(2,4-dichlorophenyl)ethanone (**5e**): yellowish crystals, m.p. 183-185 °C; ^{1}H NMR (500 MHz, DMSO-d_6) δ = 5.94 (s, 2H, CH_2), 7.65 (brs, 1H, C_6H_2), 7.71 (dd, J = 8.31 Hz, J = 1.47 Hz, 1H, C_6H_3), 7.85 (d, J = 1.47 Hz, 1H, C_6H_3), 7.99 (brs, 1H, C_6H_2), 8.12 (d, J = 8.31 Hz, 1H, C_6H_3), 8.31 (s, 1H, N=CH); ^{13}C NMR (125 MHz, DMSO-d_6) δ = 53.42, 113.28, 113.69, 114.98, 126.65, 127.64, 130.63, 131.87, 132.44, 133.13, 136.01, 137.55, 140.84, 146.39, 193.17; HRMS: calculated for $C_{15}H_9Br_2Cl_2N_2O$ $[M+H]^+$: 462.84327. Found: 462.84420.

2-(4,6-dibromo-1*H*-benzimidazol-1-yl)-1-(3,4-dichlorophenyl)ethanone (**5f**): white crystals, m.p. 190-192 °C; ^{1}H NMR (500 MHz, DMSO-d$_6$) δ = 6.06 (s, 2H, CH$_2$), 7.64 (d, J = 1.47 Hz, 1H, C$_6$H$_2$), 7.92 (d, 1H, J = 8.31 Hz, C$_6$H$_3$), 8.01-8.03 (m, 1H from C$_6$H$_3$ and 1H from C$_6$H$_2$), 8.29 (d, J = 1.96 Hz, 1H, N=CH), 8.32 (d, J = 1.96 Hz, 1H, C$_6$H$_3$); ^{13}C NMR (125 MHz, DMSO-d$_6$) δ = 51.59, 113.22, 113.75, 114.93, 126.57, 128.14, 130.18, 131.23, 131.87, 134.55, 136.18, 136.72, 140.81, 146.42, 191.37; HRMS: calculated for $C_{15}H_9Br_2Cl_2N_2O$ [M+H]$^+$: 462.84327. Found: 462.84437.

2-(4,6-dibromo-1*H*-benzimidazol-1-yl)-1-(2,4-difluorophenyl)ethanone (**5h**): white crystals, m.p. 159-160 °C; ^{1}H NMR (500 MHz, DMSO-d$_6$) δ = 5.87 (d, J = 3.42 Hz, 2H, CH$_2$), 7.33 (td, J = 8.31 Hz, J = 1.96 Hz, 1H, C$_6$H$_3$), 7.56-7.60 (m, 1H, C$_6$H$_3$), 7.63 (d, J = 1.66 Hz, 1H, C$_6$H$_2$), 8.04 (d, J = 1.66 Hz, 1H, C$_6$H$_2$), 8.04-8.09 (m, 1H, C$_6$H$_3$), 8.27 (s, 1H, N=CH); ^{13}C NMR (125 MHz, DMSO-d$_6$) δ = 54.21 (d, J = 12.72 Hz), 105.07-105.50 (m), 112.65 (dd, J = 21.52 Hz, J = 2.93 Hz), 113.13, 113.90, 114.86, 119.71 (dd, J = 12.72 Hz, J = 2.93 Hz), 126.47, 132.62 (d, J = 10.76 Hz, J = 3.91 Hz), 136.25, 140.83, 146.45, 162.70 (dd, J = 258.23 Hz, J = 13.69 Hz), 165.70 (dd, J = 255.30 Hz, J = 12.72 Hz), 189.37 (d, J = 5.87 Hz); HRMS: calculated for $C_{15}H_9Br_2F_2N_2O$ [M+H]$^+$: 430.90237. Found: 430.90263.

2-(4,6-dibromo-1*H*-benzimidazol-1-yl)-1-(2,4,6-trifluorophenyl)ethanone (**5j**): white crystals, m.p. 140-141 °C; ^{1}H NMR (500 MHz, DMSO-d$_6$) δ = 5.78 (s, 2H, CH$_2$), 7.45-7.49 (m, 1H, C$_6$H$_2$F$_3$), 7.65 (d, J = 1.96 Hz, 1H, C$_6$H$_2$), 7.96 (d, J = 1.96 Hz, 1H, C$_6$H$_2$), 8.31 (s, 1H, N=CH); ^{13}C NMR (125 MHz, DMSO-d$_6$) δ = 54.88-54.98 (m), 101.85-102.31 (m), 113.24, 113.68, 114.96, 126.62, 136.00, 140.85, 146.52, 160.71 (dd, J = 15.65 Hz, J = 9.78 Hz), 162.76 (dd, J = 16.63 Hz, J = 9.78 Hz), 163.41 (d, J = 15.65 Hz), 165.43 (d, J = 16.63 Hz), 188.46-188.51 (m); HRMS: calculated for $C_{15}H_8Br_2F_3N_2O$ [M+H]$^+$: 448.89295. Found: 448.89323.

2. ^{1}H and ^{13}C NMR of compounds 4a, 4j, 5b, 5d, 5e, 5f, 5h, 5j

2-(5,6-dibromo-1*H*-benzimidazol-1-yl)-1-phenylethanone (4a)

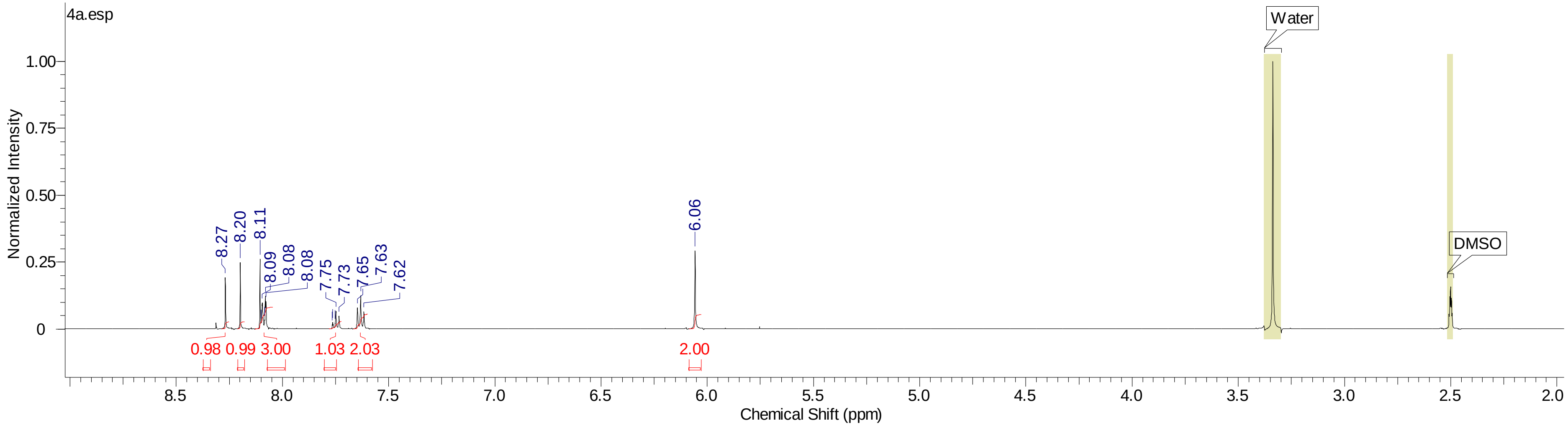

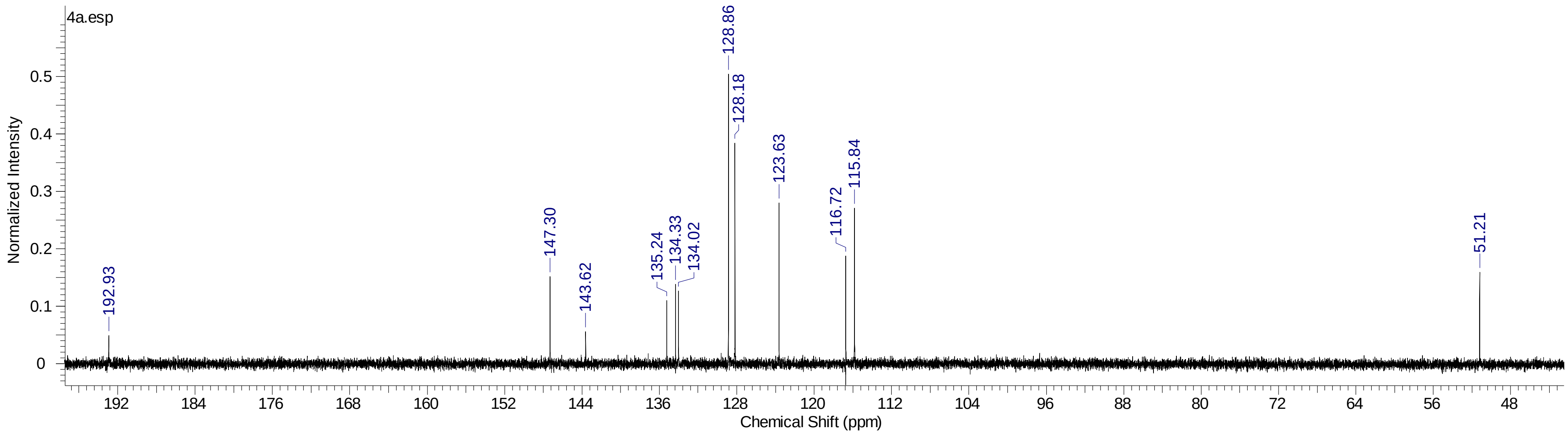

2-(5,6-dibromo-1*H*-benzimidazol-1-yl)-1-(2,4,6-trifluorophenyl)ethanone (**4j**)

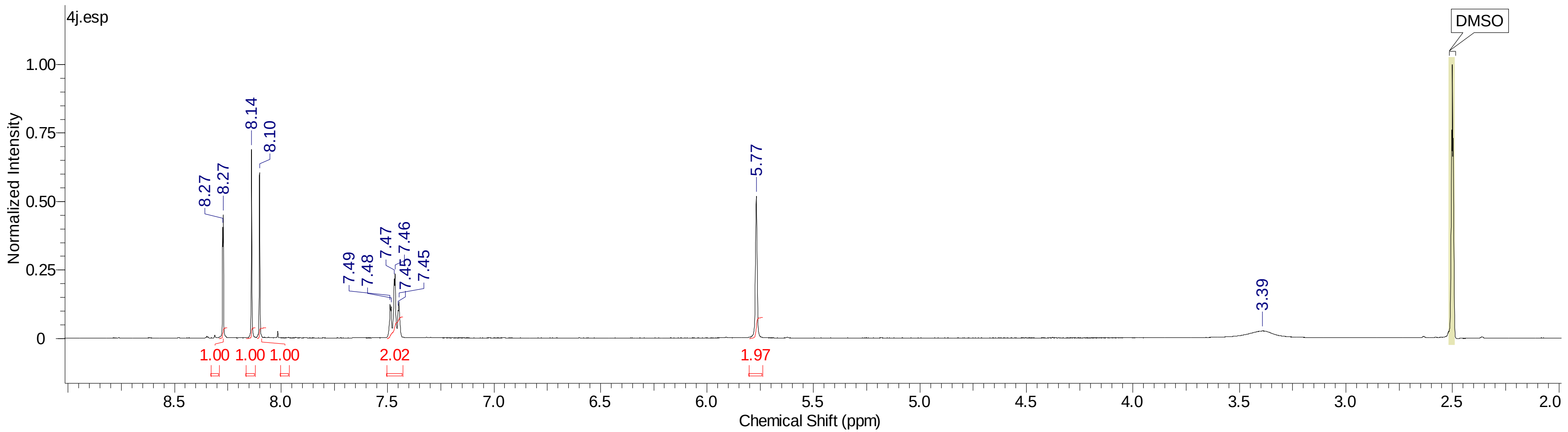

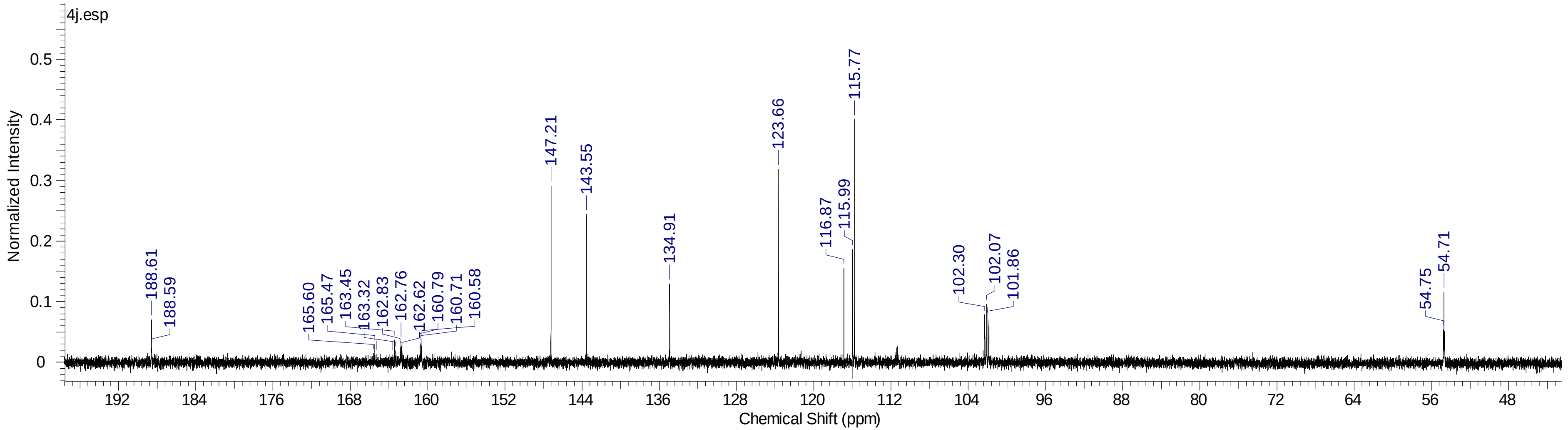

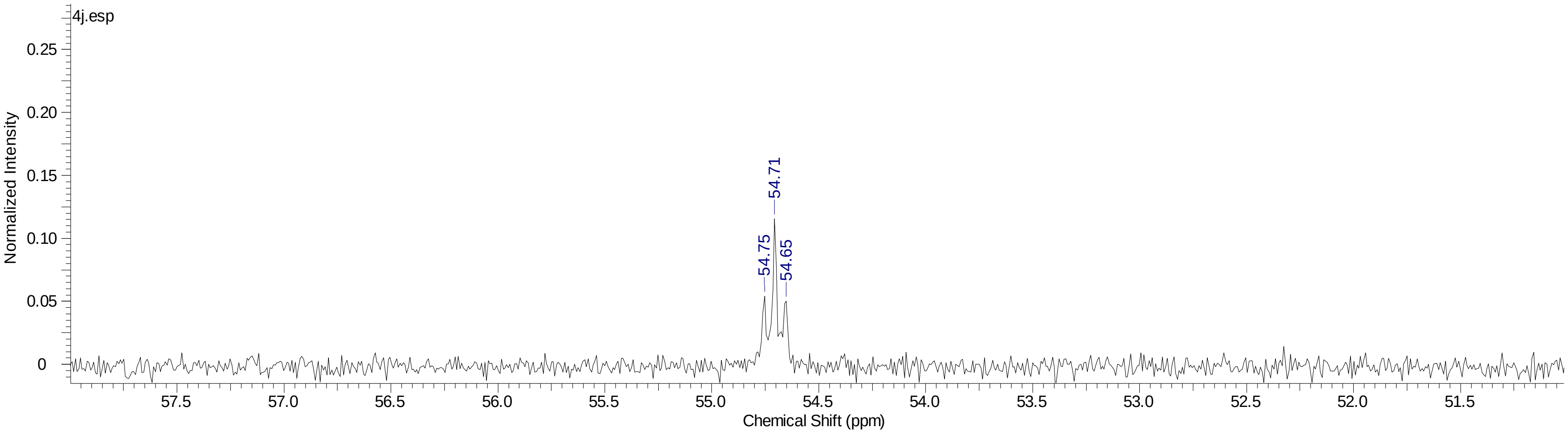
4j.esp
Normalized Intensity
Chemical Shift (ppm)
54.71
54.75
54.65

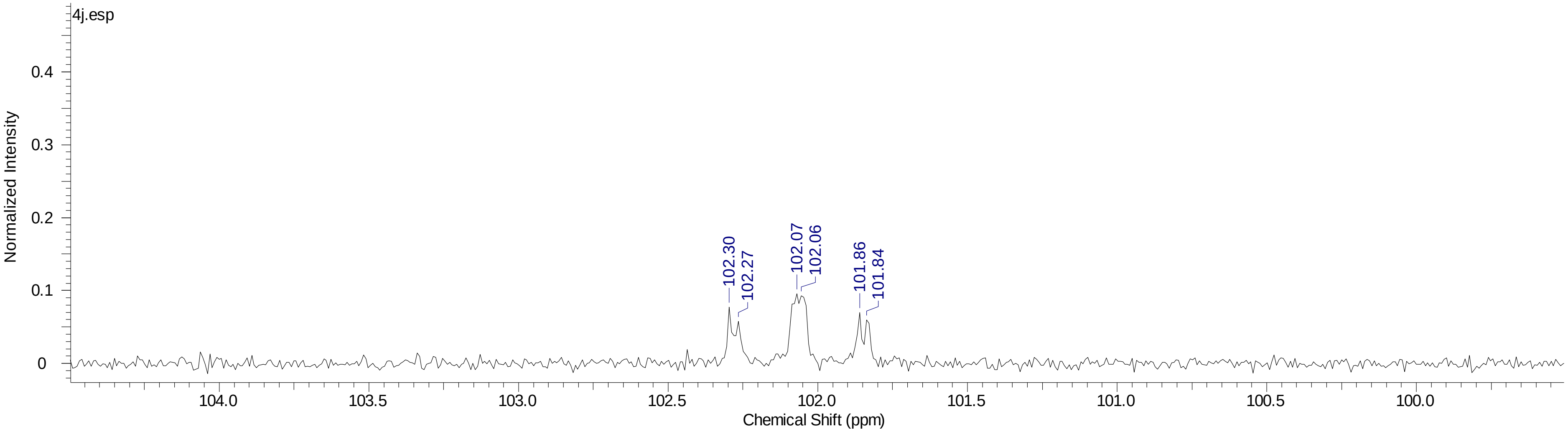
4j.esp
Normalized Intensity
Chemical Shift (ppm)
102.30
102.27
102.07
102.06
101.86
101.84

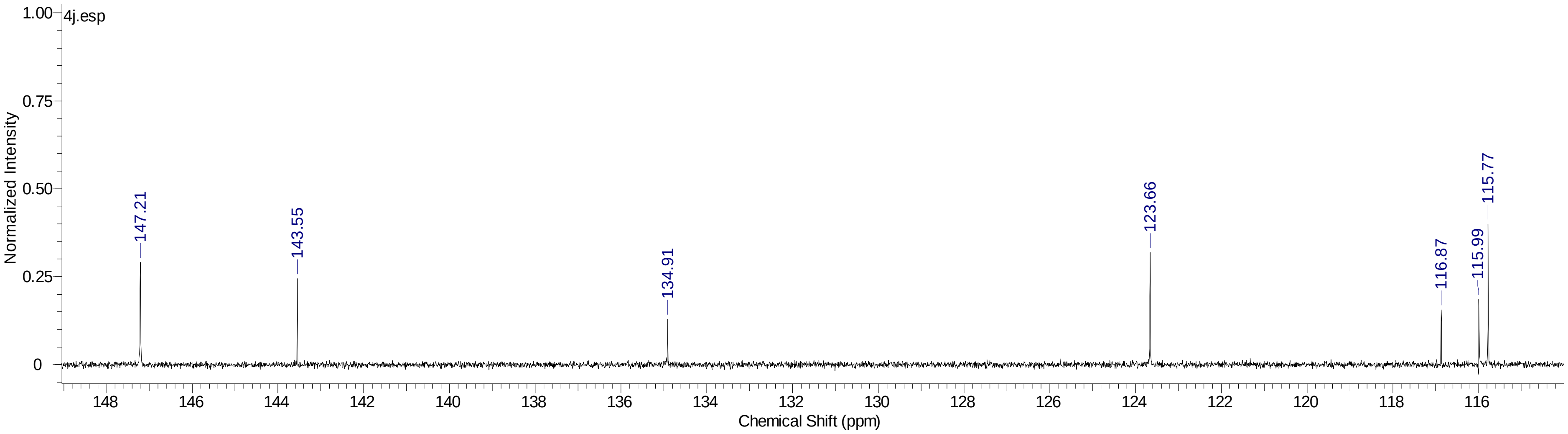
4j.esp
Normalized Intensity
1.00
0.75
0.50
0.25
0
147.21
143.55
134.91
123.66
116.87
115.99
115.77
148
146
144
142
140
138
136
134
132
130
128
126
124
122
120
118
116
Chemical Shift (ppm)

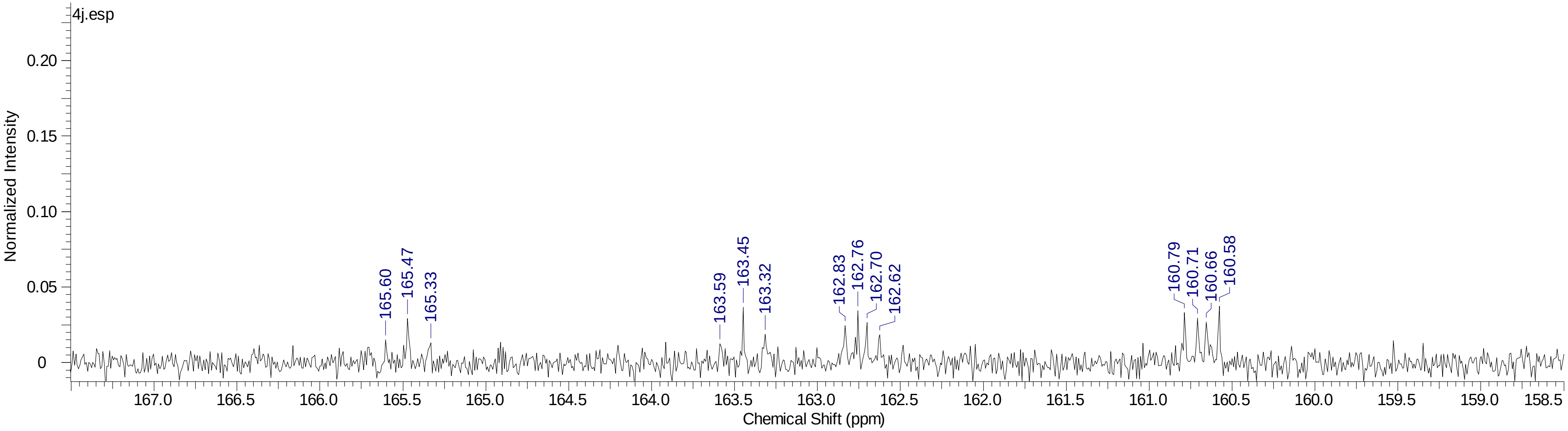
4j.esp
Normalized Intensity
0.20
0.15
0.10
0.05
0
165.60
165.47
165.33
163.59
163.45
163.32
162.83
162.76
162.70
162.62
160.79
160.71
160.66
160.58
167.0
166.5
166.0
165.5
165.0
164.5
164.0
163.5
163.0
162.5
162.0
161.5
161.0
160.5
160.0
159.5
159.0
158.5
Chemical Shift (ppm)

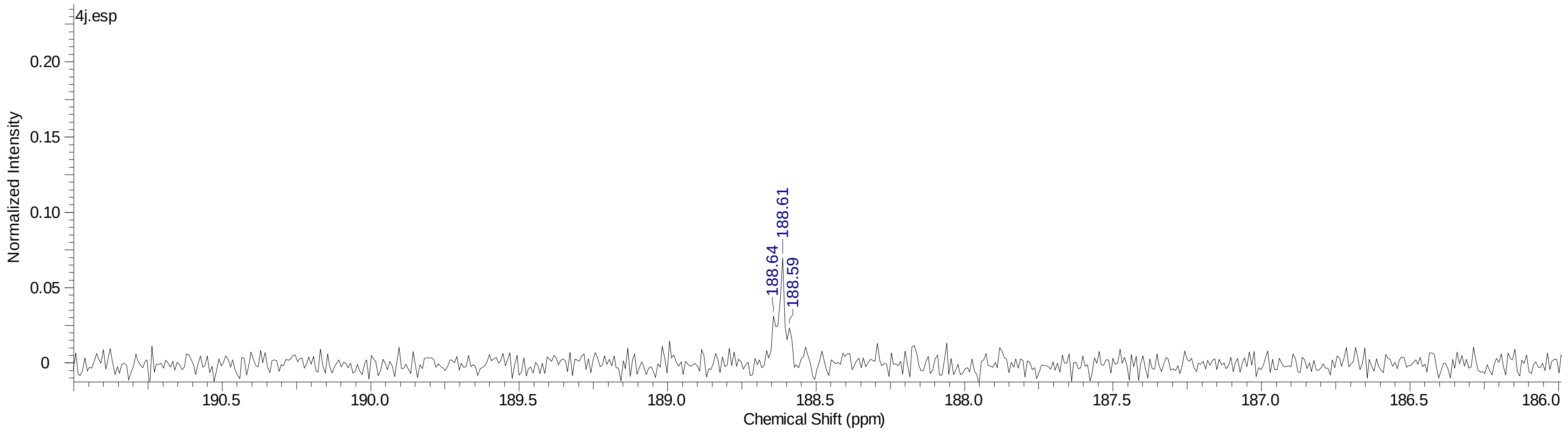

2-(4,6-dibromo-1*H*-benzimidazol-1-yl)-1-(4-fluorophenyl)ethanone (**5b**)

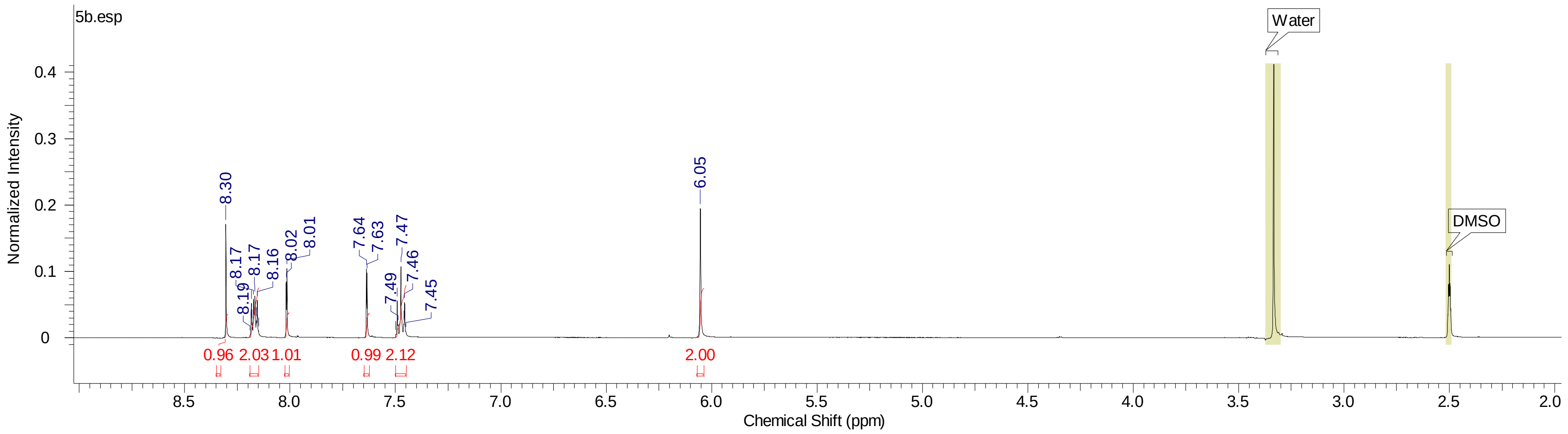

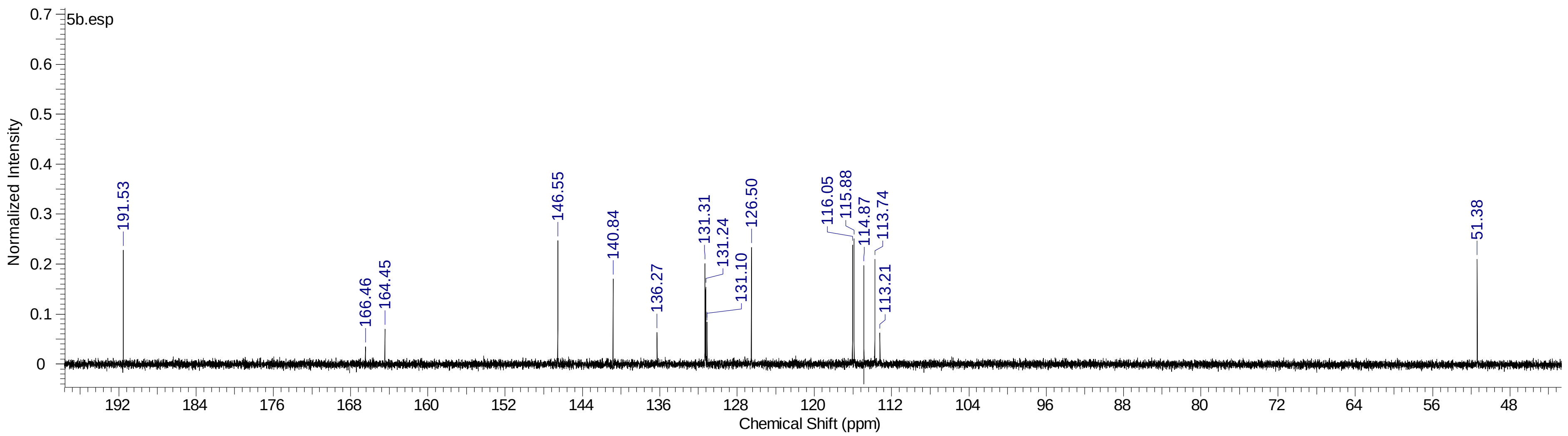
5b.esp
Normalized Intensity
Chemical Shift (ppm)
191.53
166.46
164.45
146.55
140.84
136.27
131.31
131.24
131.10
126.50
116.05
115.88
114.87
113.74
113.21
51.38

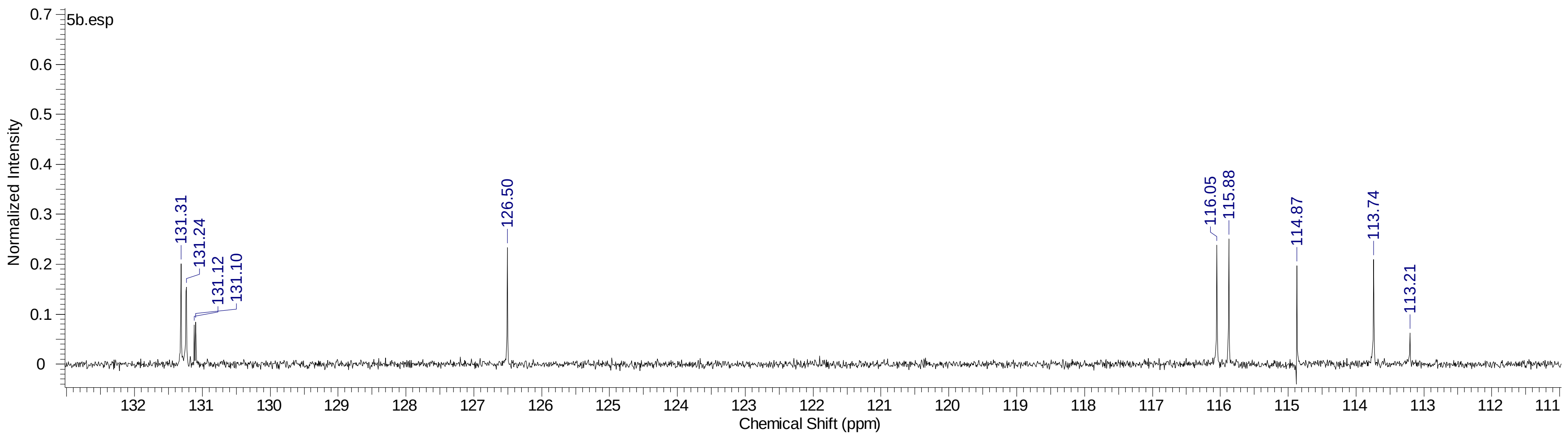
5b.esp
Normalized Intensity
Chemical Shift (ppm)
131.31
131.24
131.12
131.10
126.50
116.05
115.88
114.87
113.74
113.21

2-(4,6-dibromo-1*H*-benzimidazol-1-yl)-1-(4-bromophenyl)ethanone (**5d**)

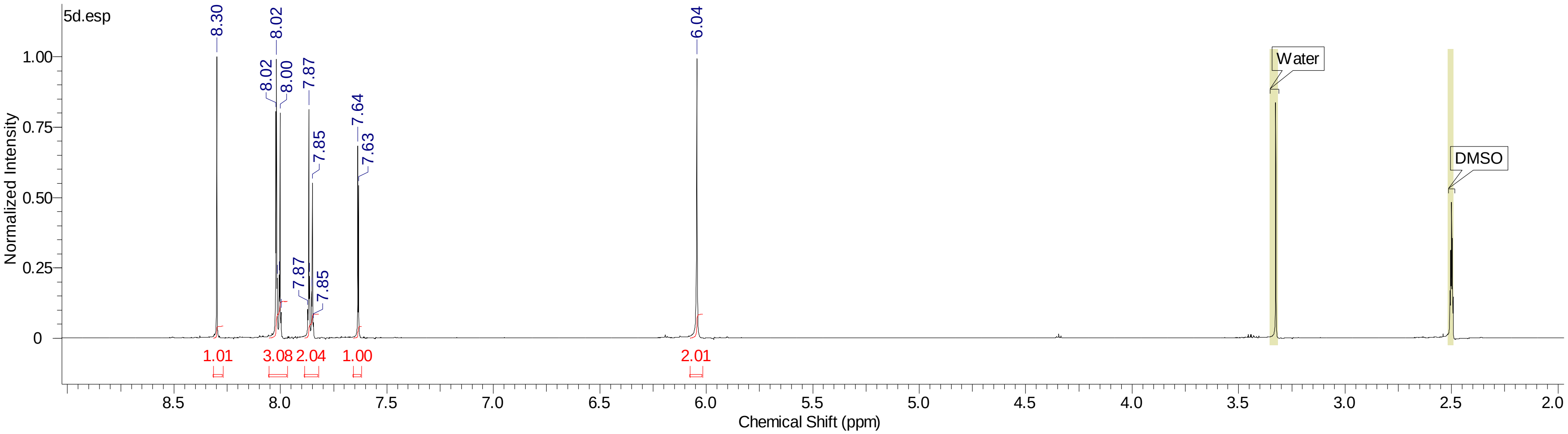

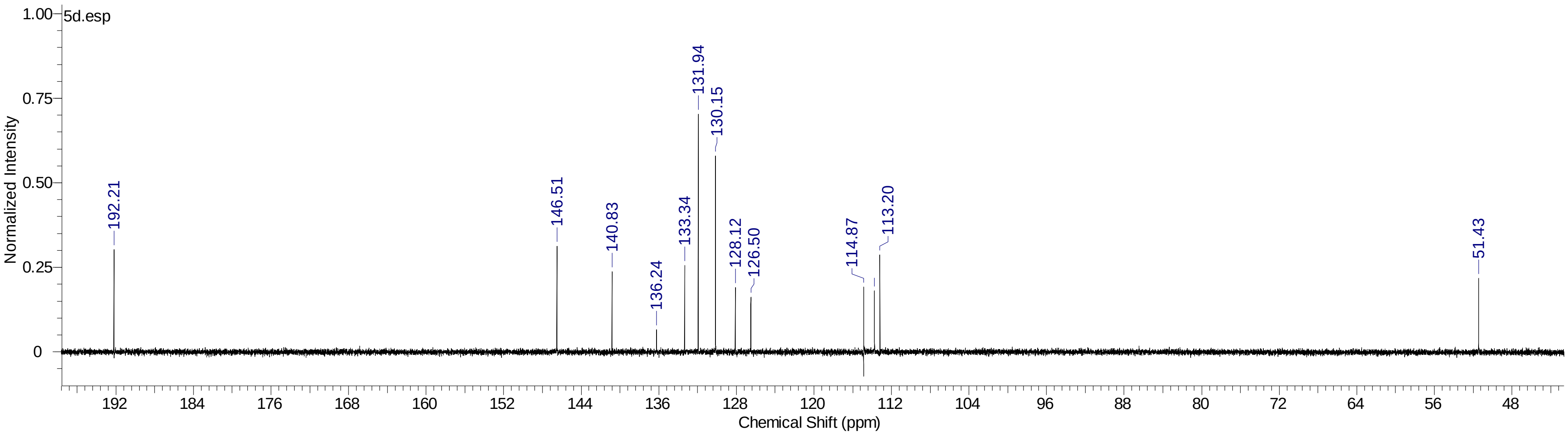

2-(4,6-dibromo-1*H*-benzimidazol-1-yl)-1-(2,4-dichlorophenyl)ethanone (5e)

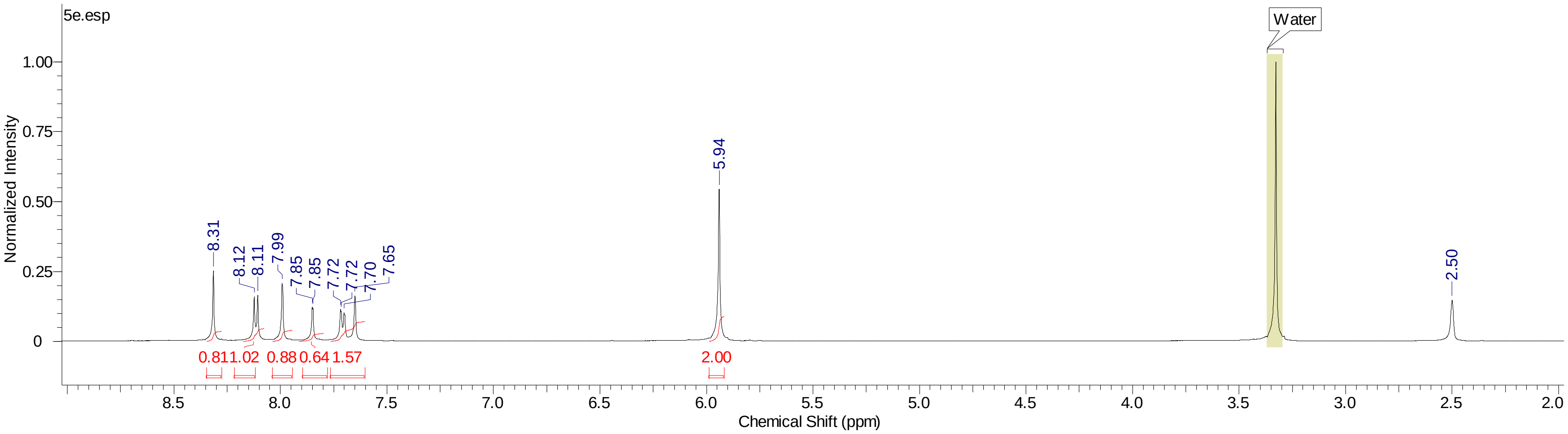

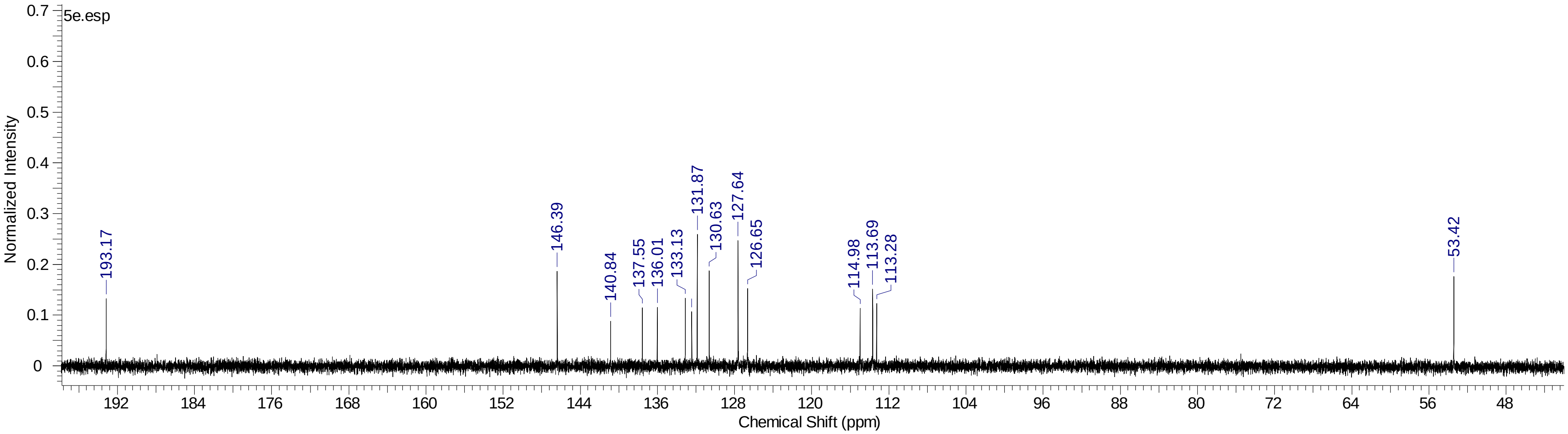

2-(4,6-dibromo-1*H*-benzimidazol-1-yl)-1-(3,4-dichlorophenyl)ethanone (**5f**)

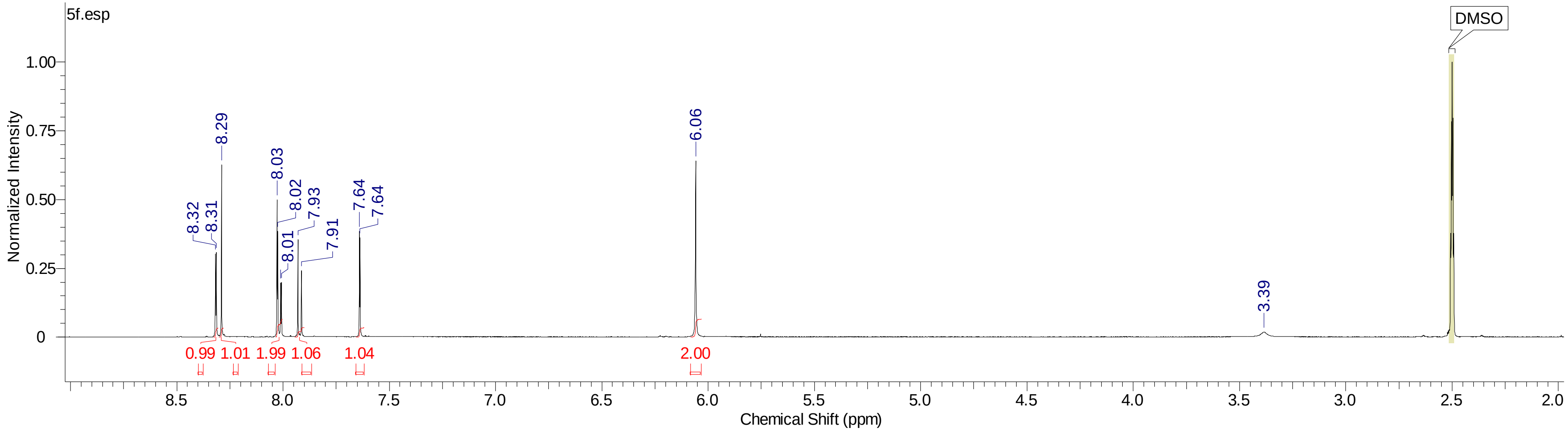

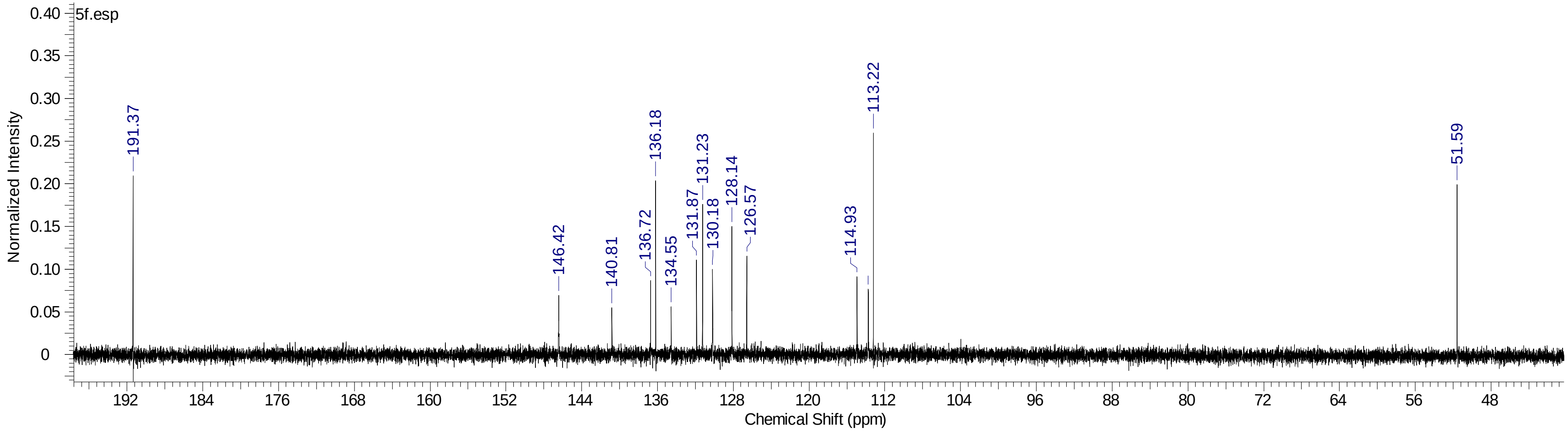

2-(4,6-dibromo-1*H*-benzimidazol-1-yl)-1-(2,4-difluorophenyl)ethanone (5h)

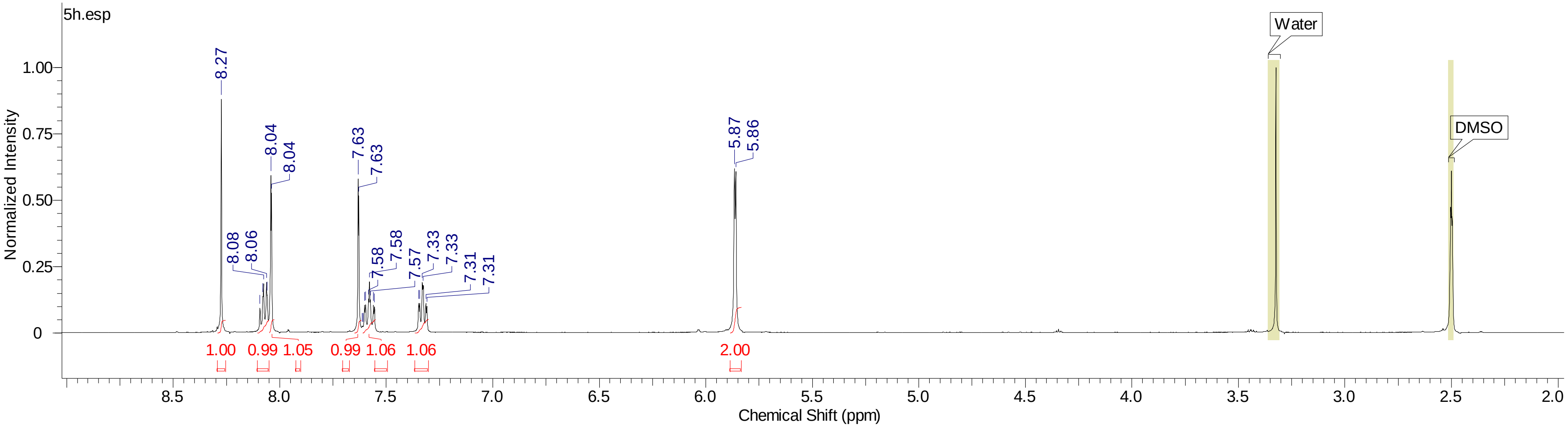

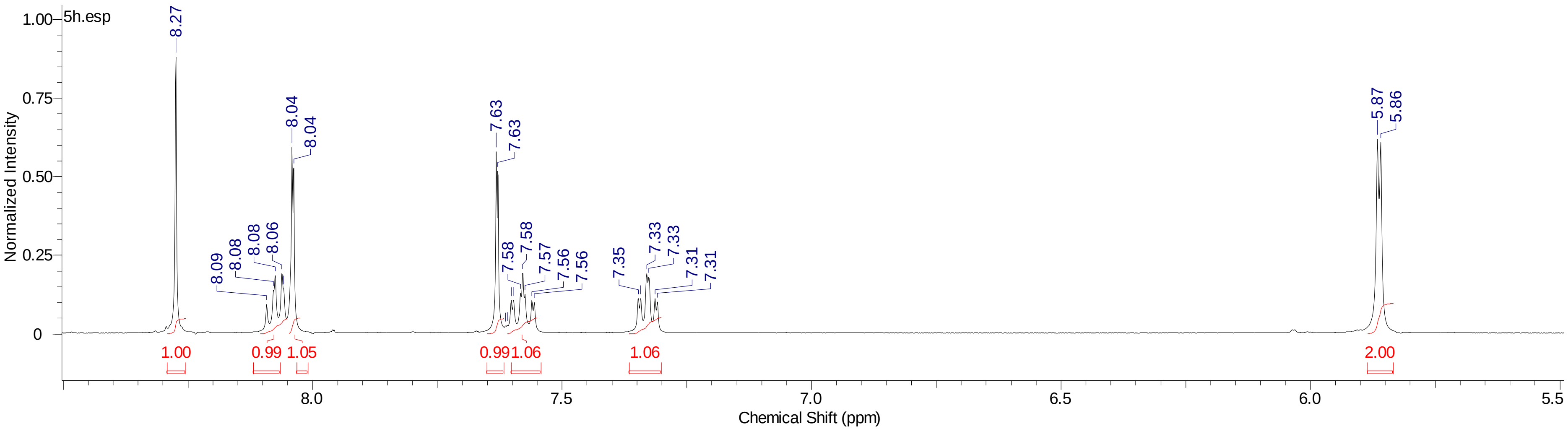

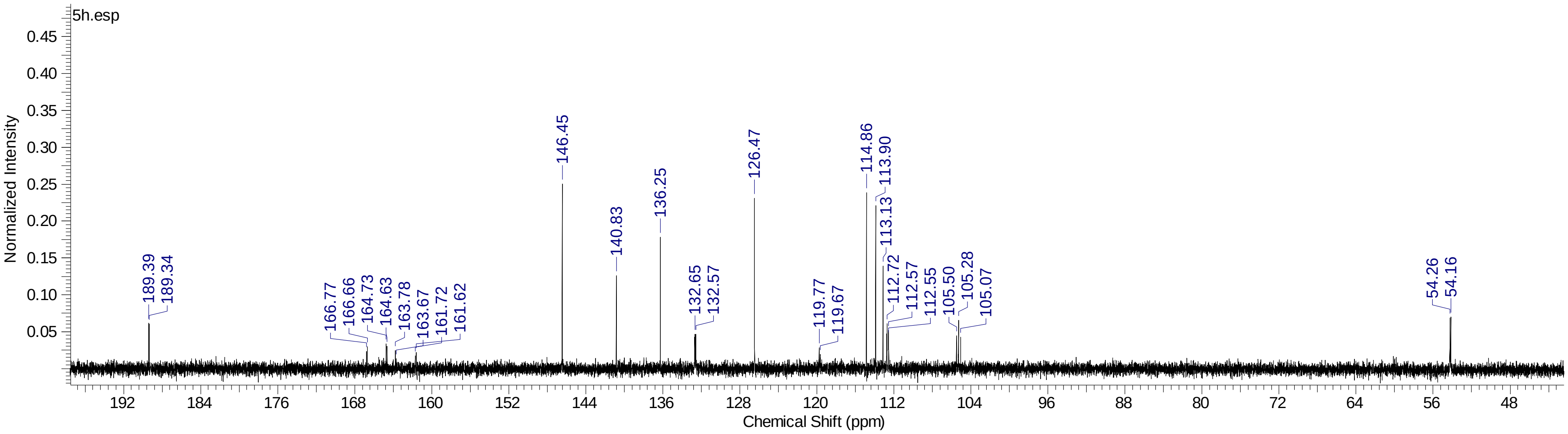
5h.esp
Normalized Intensity
Chemical Shift (ppm)
189.39
189.34
166.77
166.66
164.73
164.63
163.78
163.67
161.72
161.62
146.45
140.83
136.25
132.65
132.57
126.47
119.77
119.67
114.86
113.90
113.13
112.72
112.57
112.55
105.50
105.28
105.07
54.26
54.16

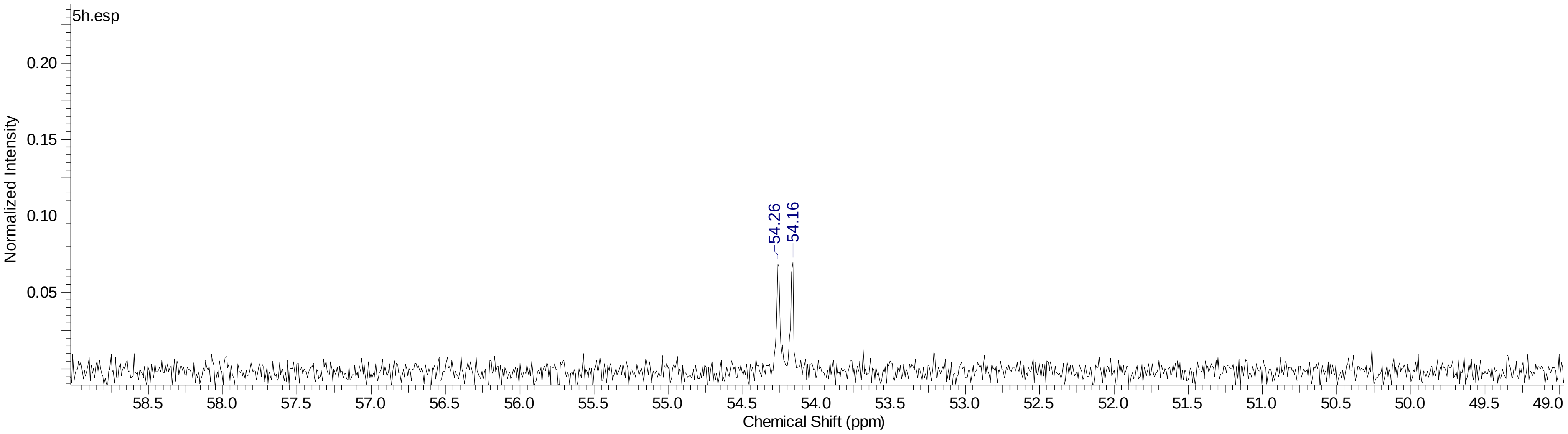
5h.esp
Normalized Intensity
Chemical Shift (ppm)
54.26
54.16

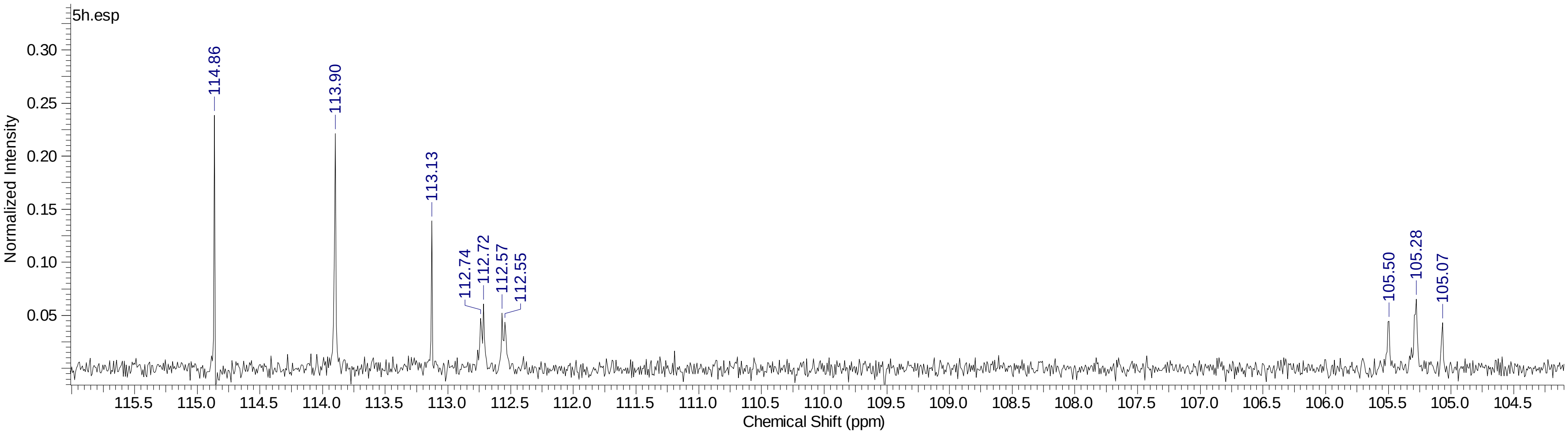
5h.esp
Normalized Intensity
0.30
0.25
0.20
0.15
0.10
0.05
114.86
113.90
113.13
112.74
112.72
112.57
112.55
105.50
105.28
105.07
115.5
115.0
114.5
114.0
113.5
113.0
112.5
112.0
111.5
111.0
110.5
110.0
109.5
109.0
108.5
108.0
107.5
107.0
106.5
106.0
105.5
105.0
104.5
Chemical Shift (ppm)

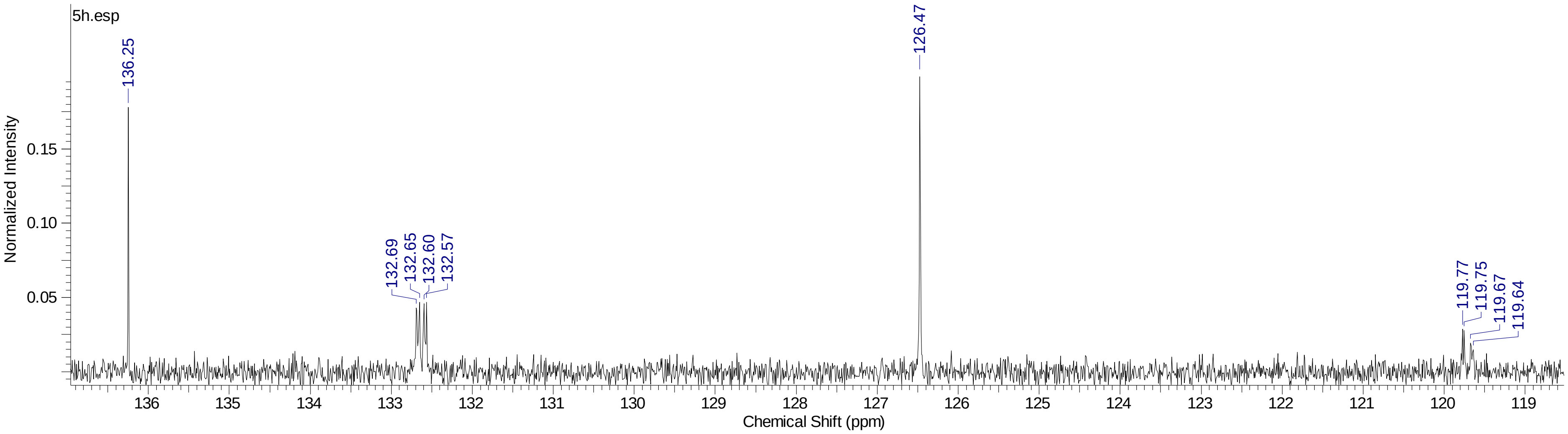
5h.esp
Normalized Intensity
0.15
0.10
0.05
136.25
132.69
132.65
132.60
132.57
126.47
119.77
119.75
119.67
119.64
136
135
134
133
132
131
130
129
128
127
126
125
124
123
122
121
120
119
Chemical Shift (ppm)

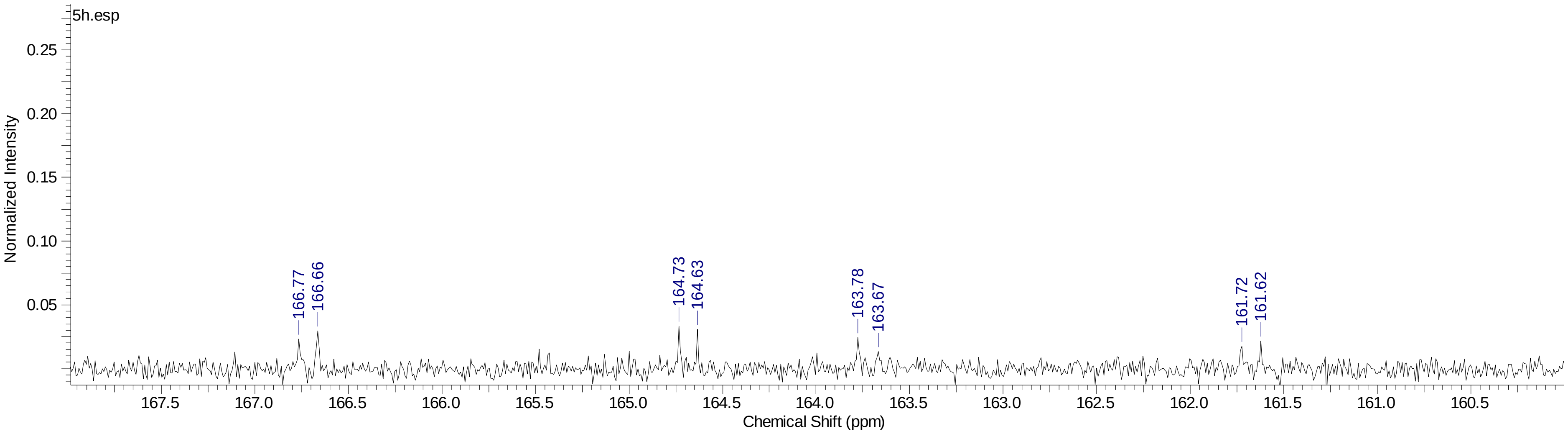
5h.esp
Normalized Intensity
0.25
0.20
0.15
0.10
0.05
166.77
166.66
164.73
164.63
163.78
163.67
161.72
161.62
167.5 167.0 166.5 166.0 165.5 165.0 164.5 164.0 163.5 163.0 162.5 162.0 161.5 161.0 160.5
Chemical Shift (ppm)

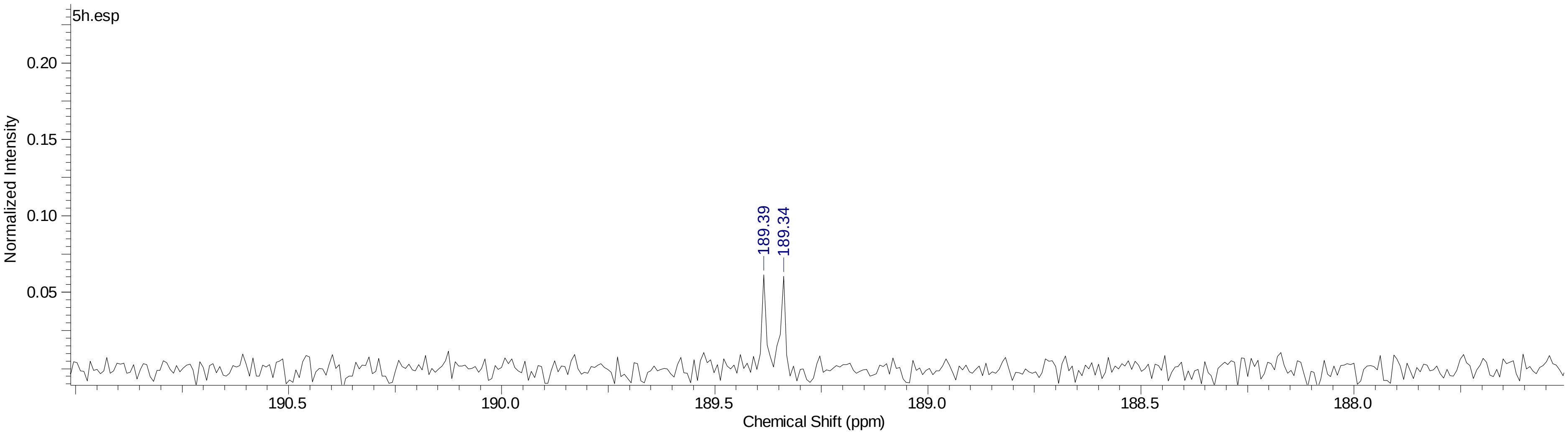
5h.esp
Normalized Intensity
0.20
0.15
0.10
0.05
189.39
189.34
190.5 190.0 189.5 189.0 188.5 188.0
Chemical Shift (ppm)

2-(4,6-dibromo-1*H*-benzimidazol-1-yl)-1-(2,4,6-trifluorophenyl)ethanone (5j)

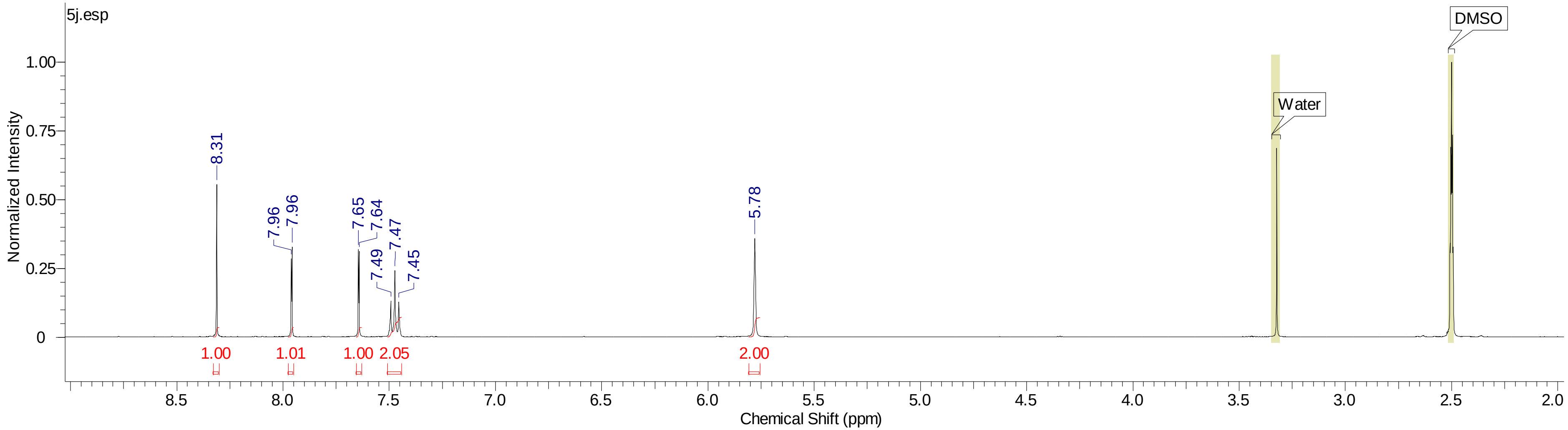

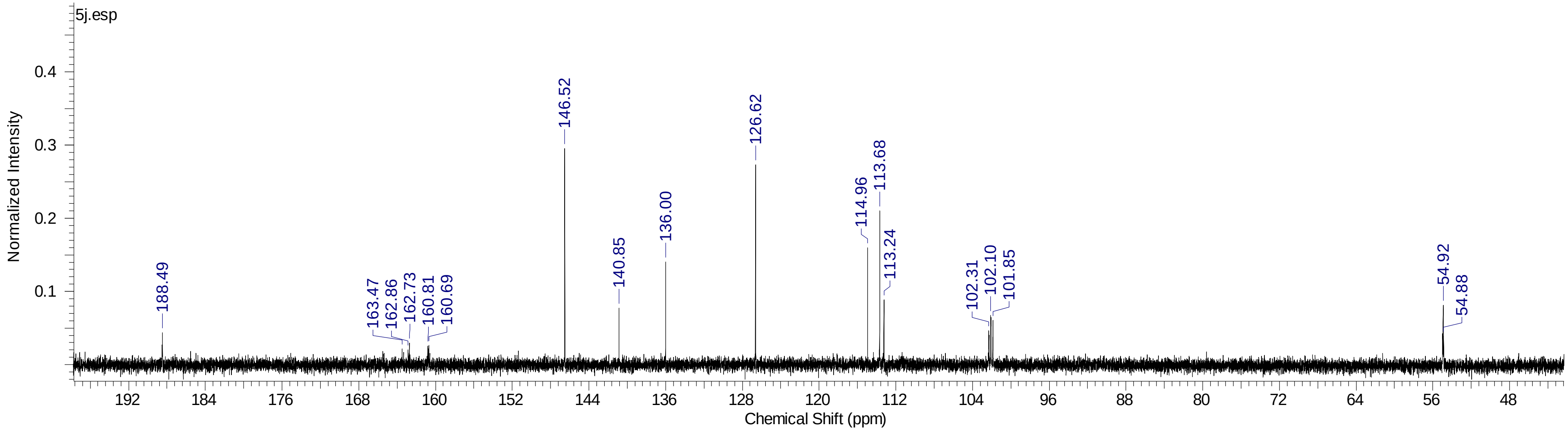

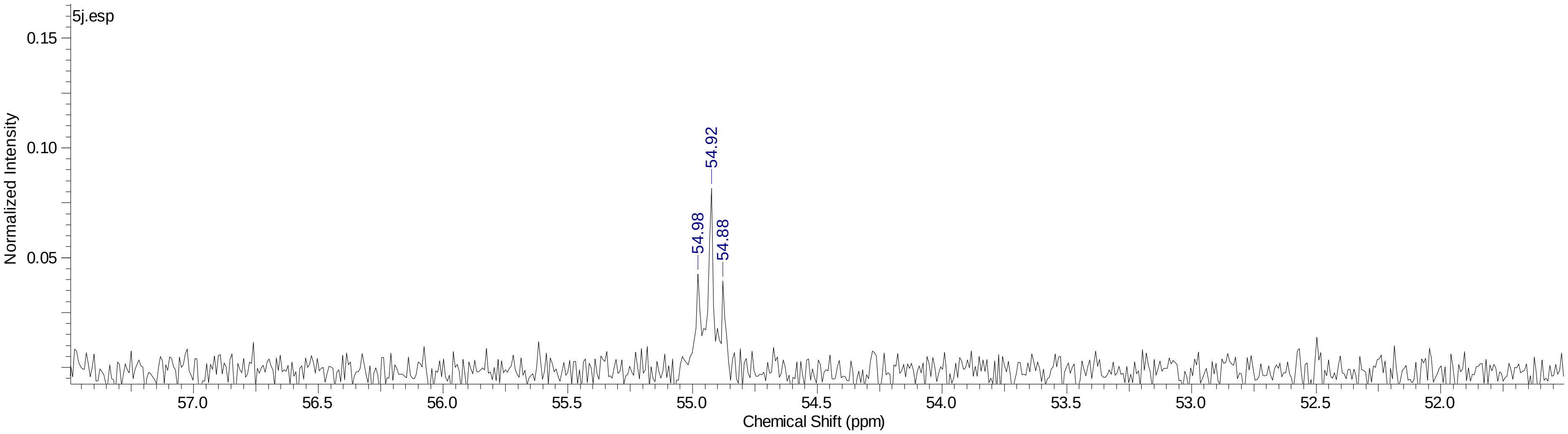
5j.esp
Normalized Intensity
0.15
0.10
0.05
54.98
54.92
54.88
57.0
56.5
56.0
55.5
55.0
54.5
54.0
53.5
53.0
52.5
52.0
Chemical Shift (ppm)

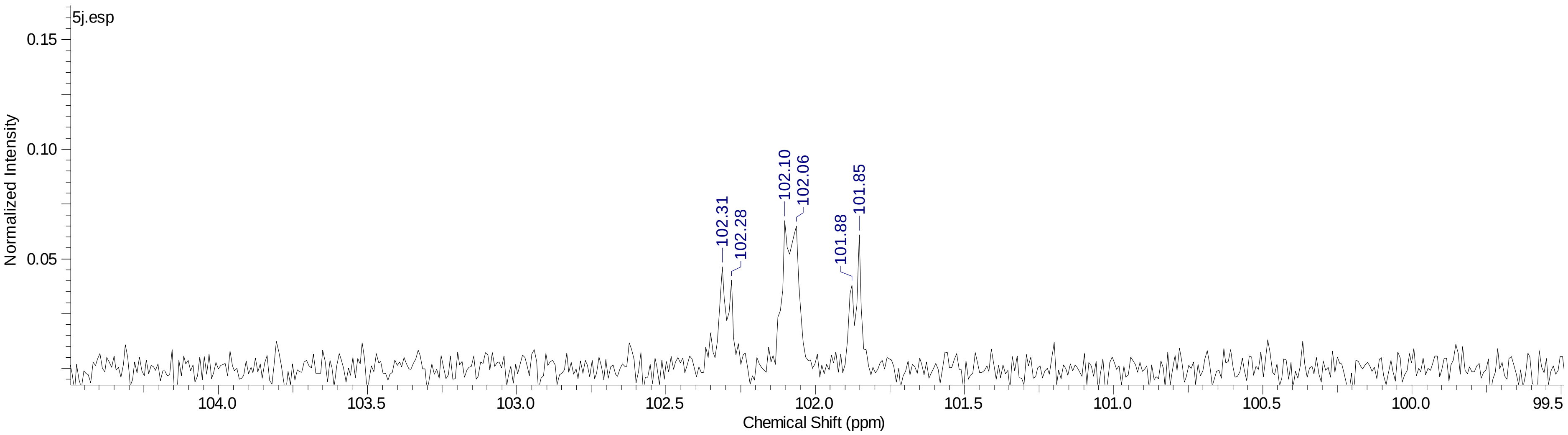
5j.esp
Normalized Intensity
0.15
0.10
0.05
102.31
102.28
102.10
102.06
101.88
101.85
104.0
103.5
103.0
102.5
102.0
101.5
101.0
100.5
100.0
99.5
Chemical Shift (ppm)

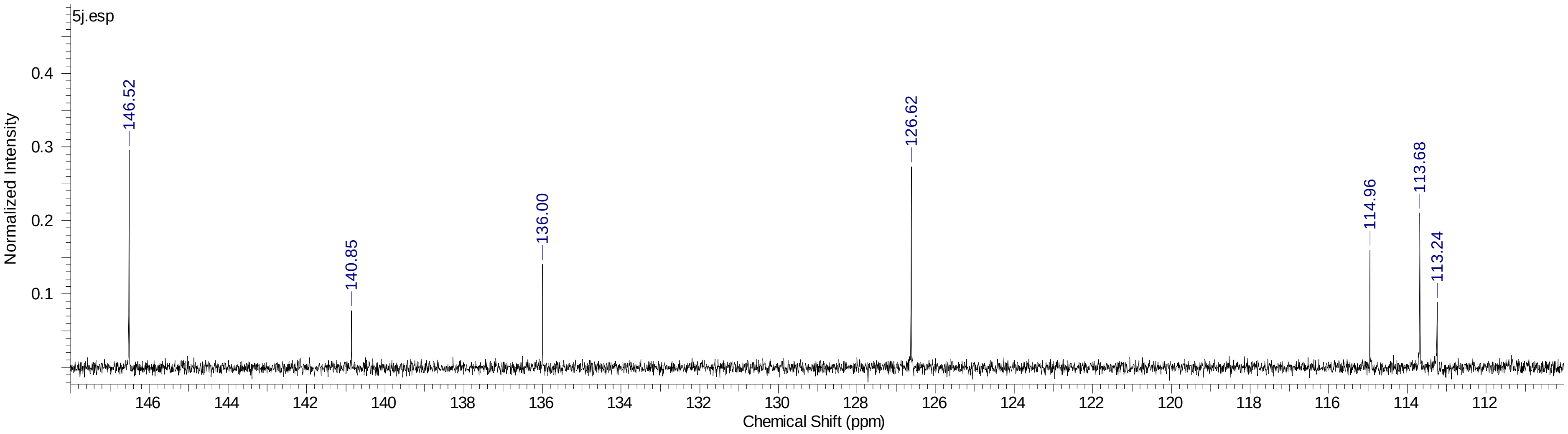
5j.esp
Normalized Intensity
Chemical Shift (ppm)
146.52
140.85
136.00
126.62
114.96
113.68
113.24

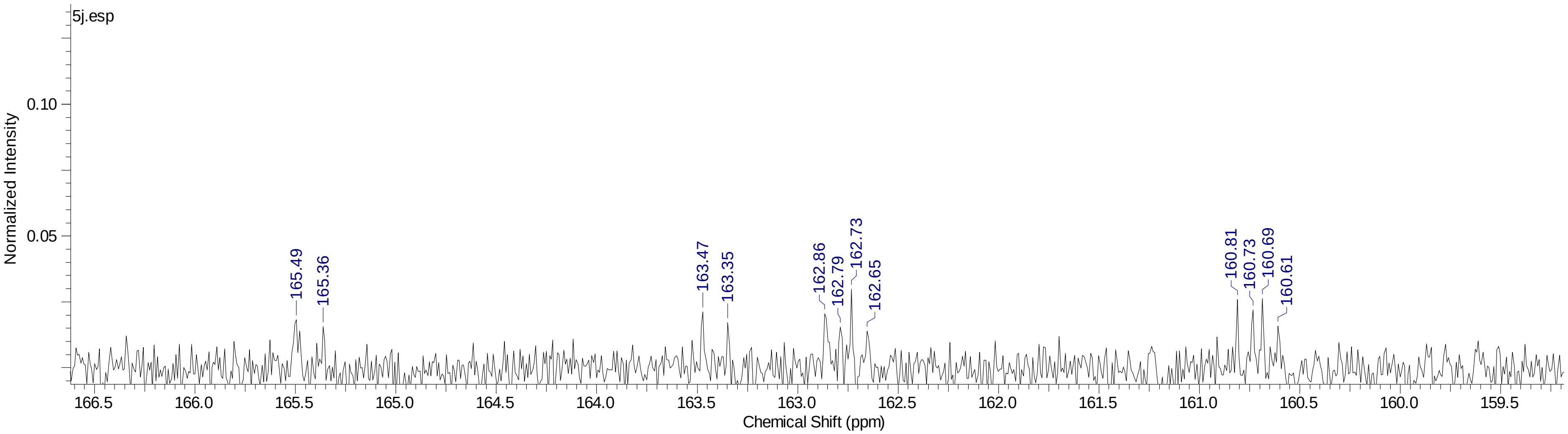
5j.esp
Normalized Intensity
Chemical Shift (ppm)
165.49
165.36
163.47
163.35
162.86
162.79
162.73
162.65
160.81
160.73
160.69
160.61

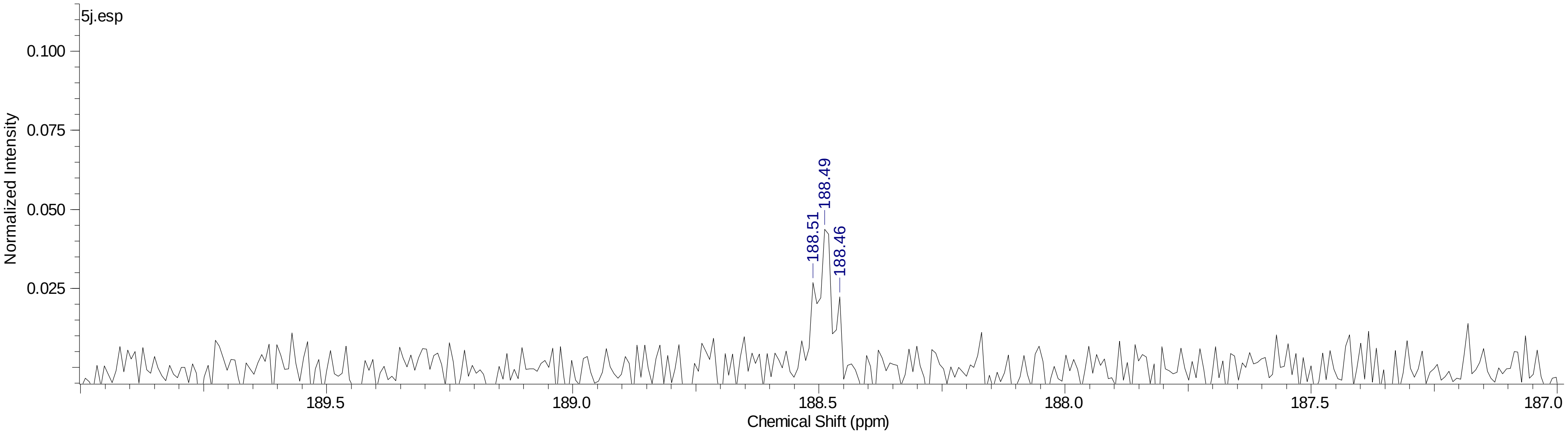
5j.esp
0.100
0.075
0.050
0.025
Normalized Intensity
188.51
188.49
188.46
189.5
189.0
188.5
188.0
187.5
187.0
Chemical Shift (ppm)

3. HR-MS of compounds 4a, 4j, 5b, 5d, 5e, 5f, 5h, 5j

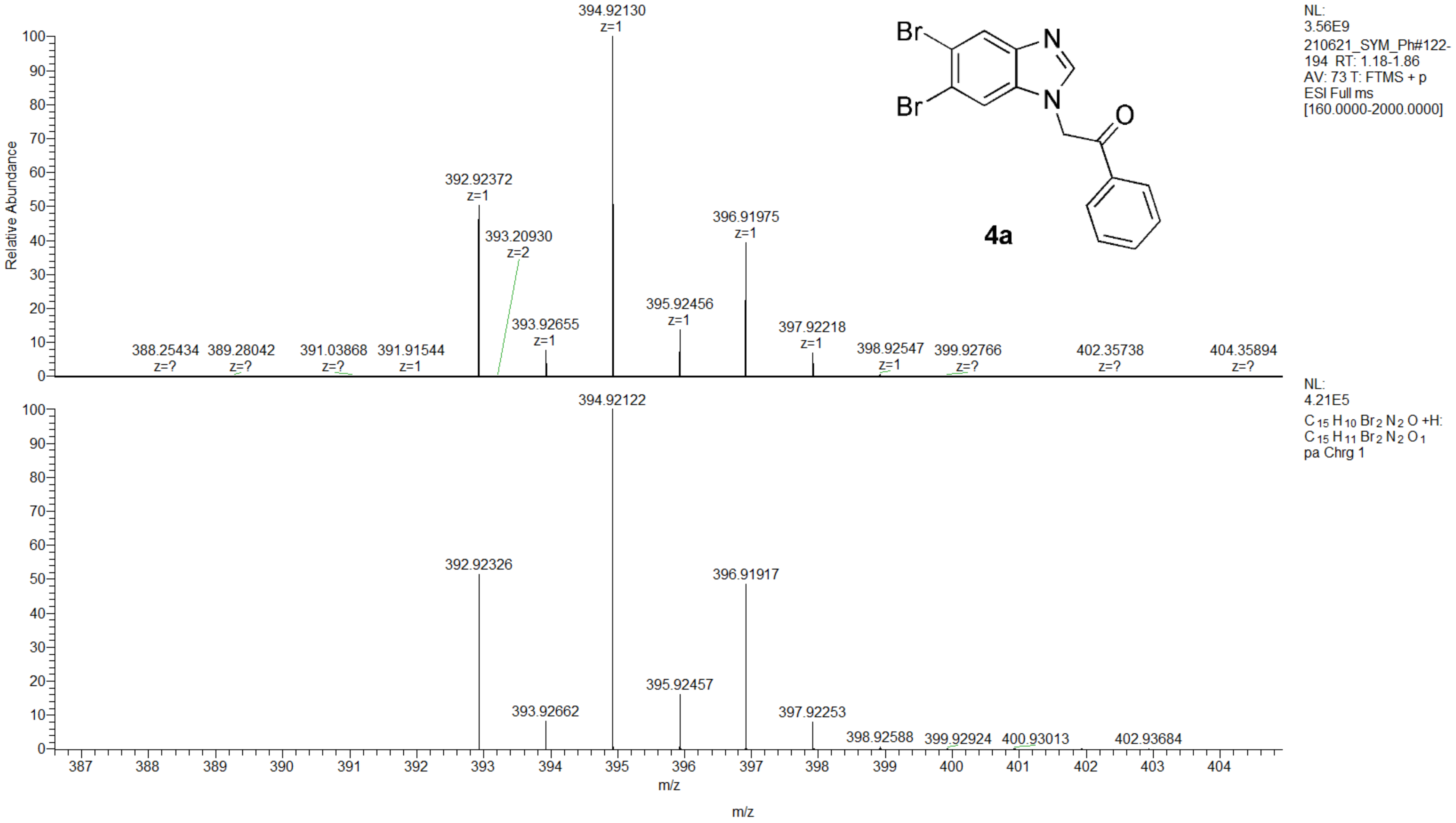

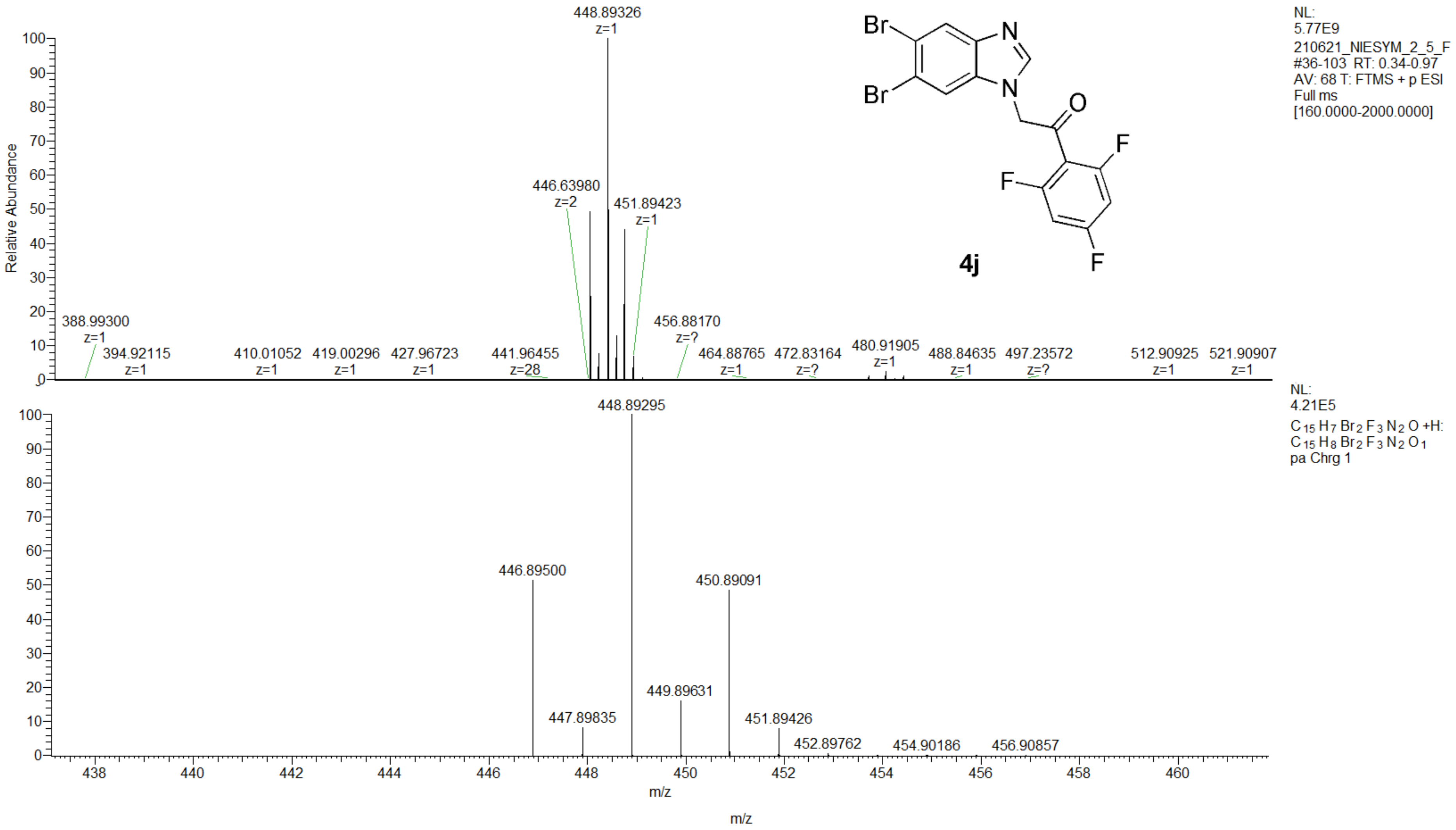
448.89326 z=1
446.63980 z=2
451.89423 z=1
388.99300 z=1
394.92115 z=1
410.01052 z=1
419.00296 z=1
427.96723 z=1
441.96455 z=28
456.88170 z=?
464.88765 z=1
472.83164 z=?
480.91905 z=1
488.84635 z=1
497.23572 z=?
512.90925 z=1
521.90907 z=1
Relative Abundance
NL: 5.77E9
210621_NIESYM_2_5_F #36-103 RT: 0.34-0.97 AV: 68 T: FTMS + p ESI Full ms [160.0000-2000.0000]
Br
Br
N
N
O
F
F
F
4j
448.89295
446.89500
450.89091
449.89631
447.89835
451.89426
452.89762
454.90186
456.90857
NL: 4.21E5
C 15 H 7 Br 2 F 3 N 2 O +H: C 15 H 8 Br 2 F 3 N 2 O 1 pa Chrg 1
m/z
m/z

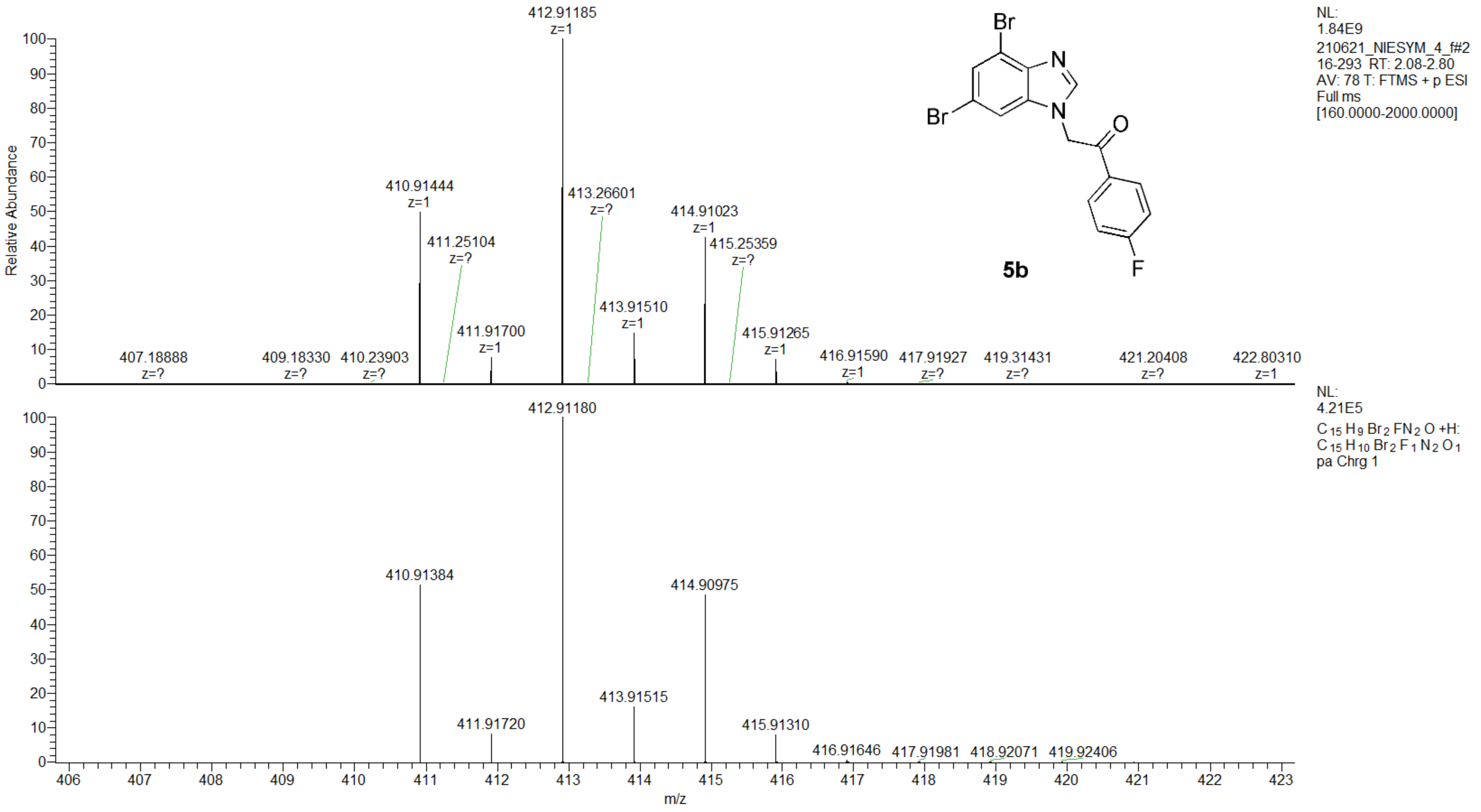

412.91185 z=1
410.91444 z=1
411.25104 z=?
413.26601 z=?
414.91023 z=1
415.25359 z=?
411.91700 z=1
413.91510 z=1
415.91265 z=1
407.18888 z=?
409.18330 z=?
410.23903 z=?
416.91590 z=1
417.91927 z=?
419.31431 z=?
421.20408 z=?
422.80310 z=1
Relative Abundance
NL: 1.84E9
210621_NIESYM_4_f#2 16-293 RT: 2.08-2.80 AV: 78 T: FTMS + p ESI Full ms [160.0000-2000.0000]
5b
412.91180
410.91384
414.90975
413.91515
411.91720
415.91310
416.91646
417.91981
418.92071
419.92406
NL: 4.21E5
C15 H9 Br2 FN2 O +H: C15 H10 Br2 F1 N2 O1 pa Chrg 1
m/z

m/z

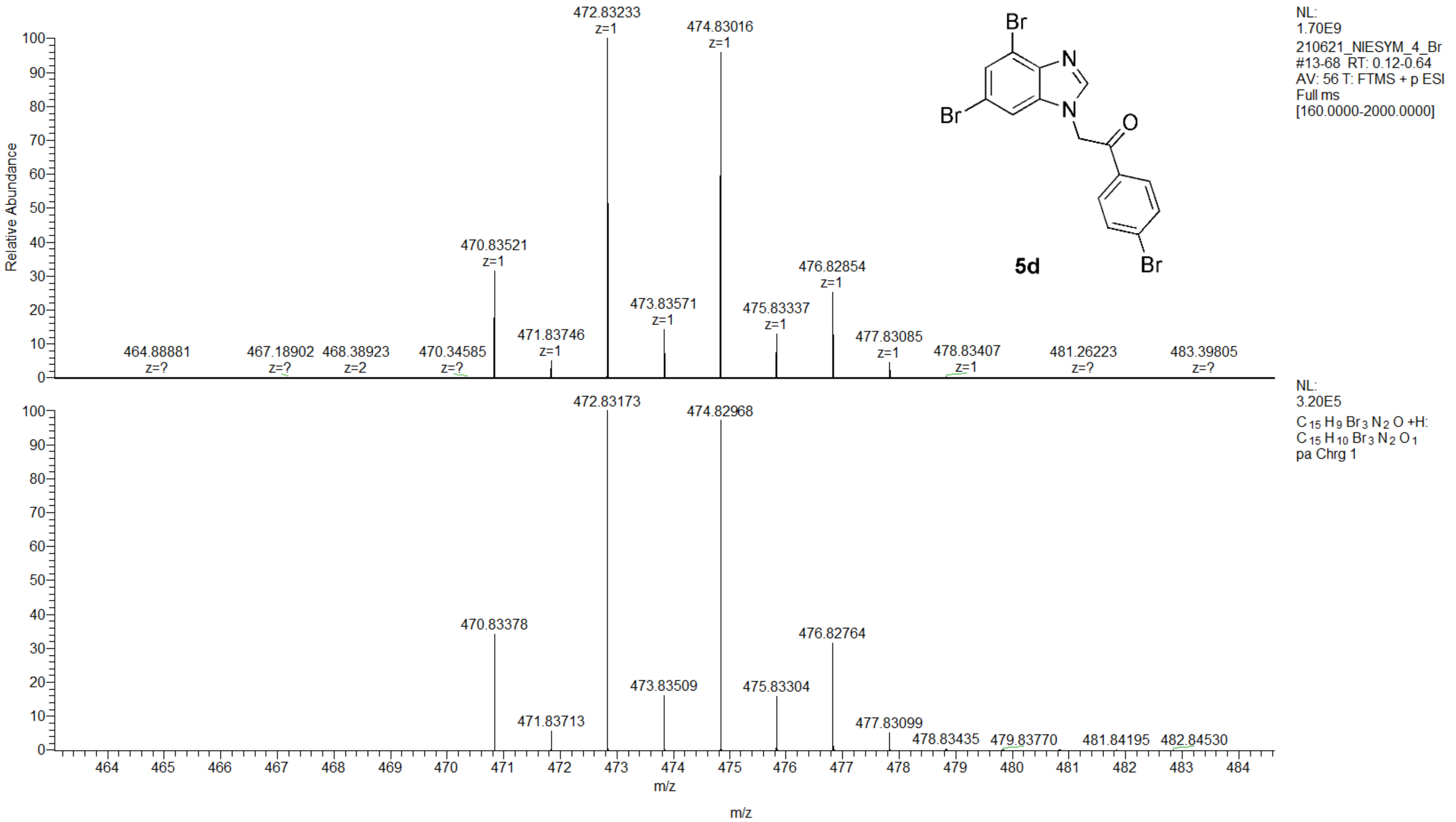
472.83233
z=1
474.83016
z=1
470.83521
z=1
476.82854
z=1
473.83571
z=1
475.83337
z=1
471.83746
z=1
477.83085
z=1
464.88881
z=?
467.18902
z=?
468.38923
z=2
470.34585
z=?
478.83407
z=1
481.26223
z=?
483.39805
z=?
Relative Abundance
Br
N
N
O
Br
Br
5d
NL:
1.70E9
210621_NIESYM_4_Br
#13-68 RT: 0.12-0.64
AV: 56 T: FTMS + p ESI
Full ms
[160.0000-2000.0000]
472.83173
474.82968
470.83378
476.82764
473.83509
475.83304
471.83713
477.83099
478.83435
479.83770
481.84195
482.84530
NL:
3.20E5
C 15 H 9 Br 3 N 2 O +H:
C 15 H 10 Br 3 N 2 O 1
pa Chrg 1
m/z
m/z

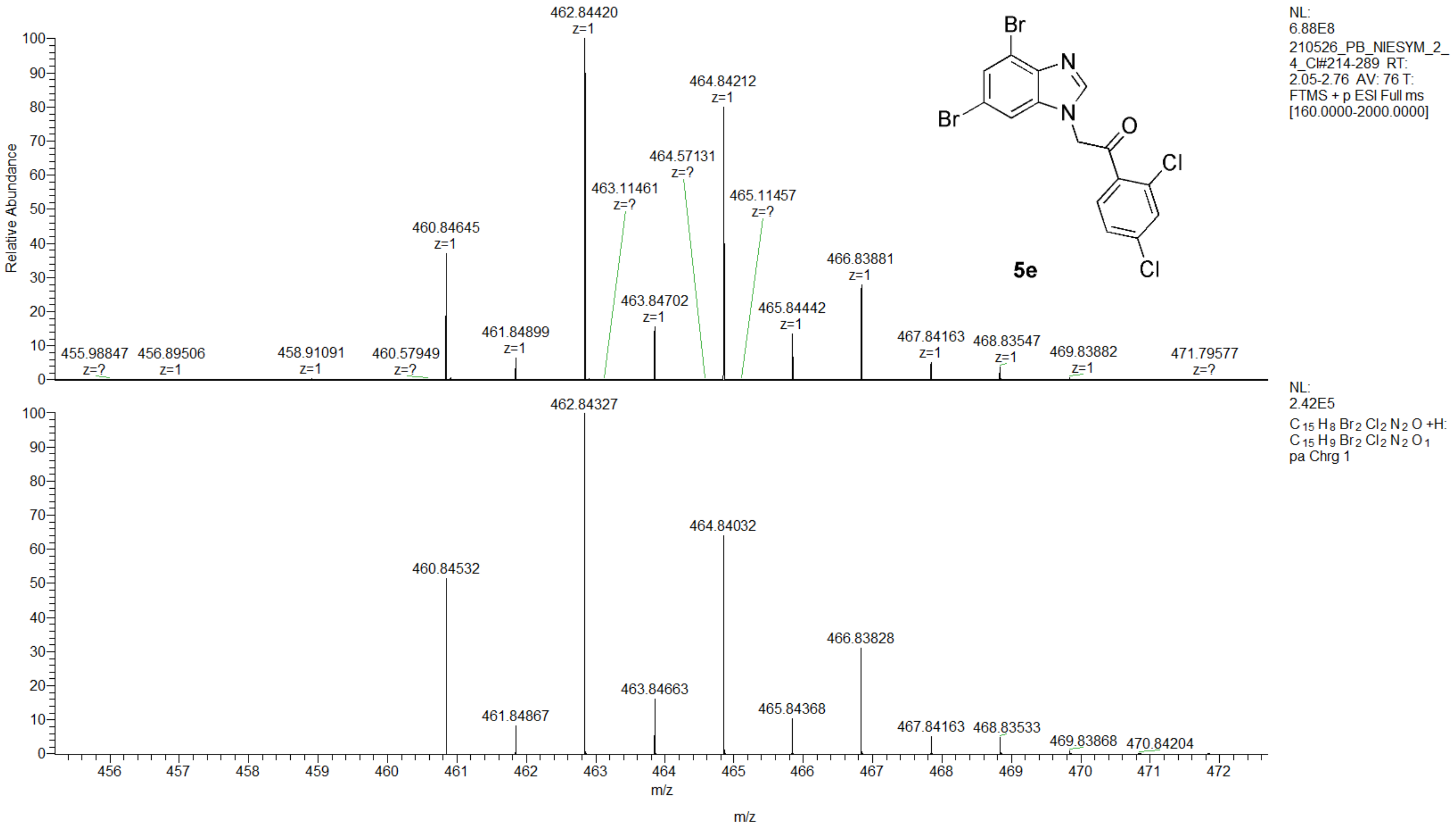

NL:
6.88E8
210526_PB_NIESYM_2_4_CI#214-289 RT: 2.05-2.76 AV: 76 T: FTMS + p ESI Full ms [160.0000-2000.0000]
NL:
2.42E5
C 15 H8 Br2 Cl2 N2 O +H: C 15 H9 Br2 Cl2 N2 O1
pa Chrg 1
Relative Abundance
m/z
5e
462.84420
464.84212
460.84645
466.83881
462.84327
464.84032
460.84532
466.83828

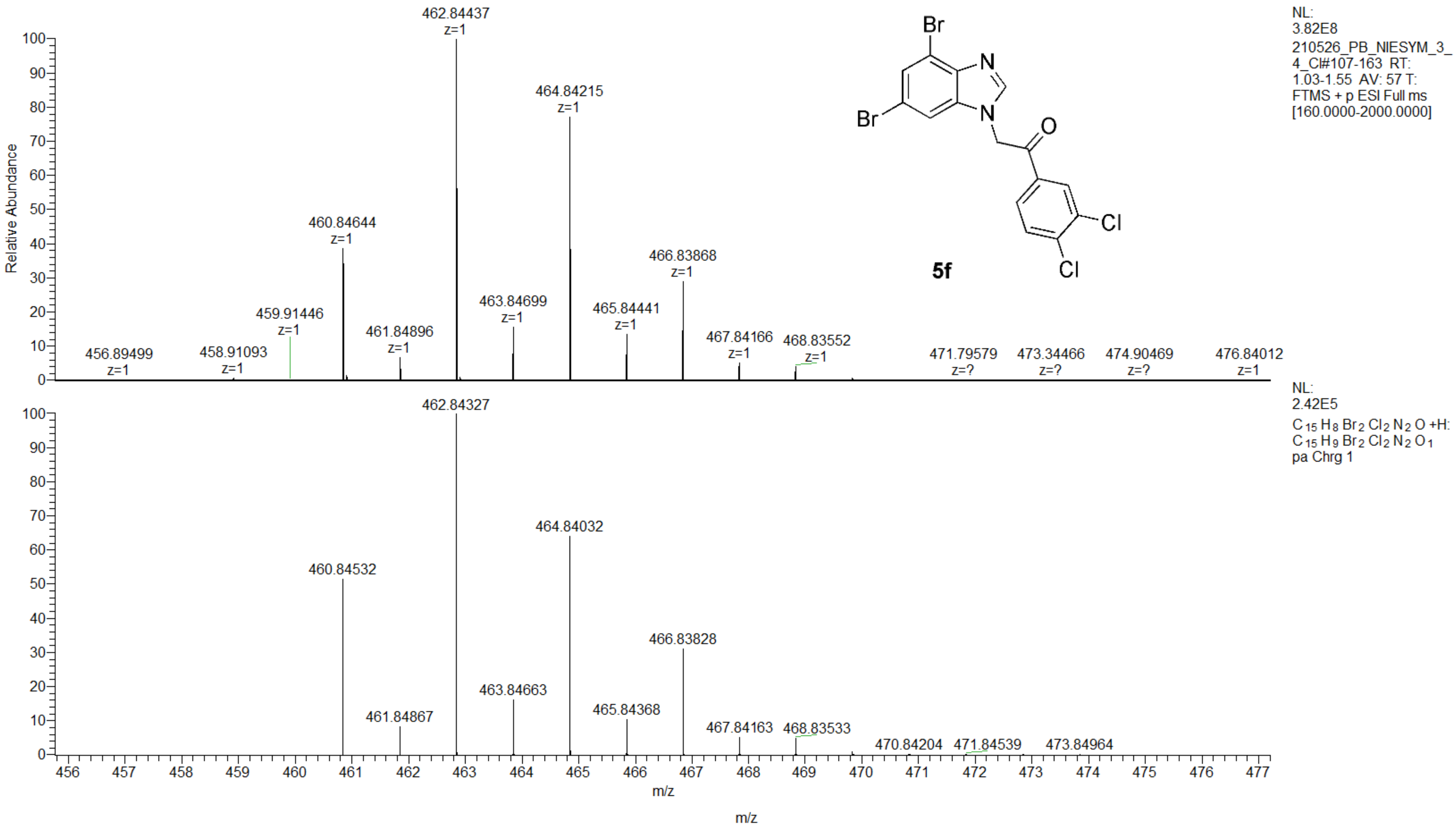

NL:
3.82E8
210526_PB_NIESYM_3_4_CI#107-163 RT: 1.03-1.55 AV: 57 T: FTMS + p ESI Full ms [160.0000-2000.0000]
462.84437 z=1
464.84215 z=1
460.84644 z=1
466.83868 z=1
463.84699 z=1
465.84441 z=1
459.91446 z=1
461.84896 z=1
467.84166 z=1
468.83552 z=1
456.89499 z=1
458.91093 z=1
471.79579 z=?
473.34466 z=?
474.90469 z=?
476.84012 z=1
Br
N
N
O
Cl
Cl
5f
NL:
2.42E5
C 15 H 8 Br 2 Cl 2 N 2 O +H:
C 15 H 9 Br 2 Cl 2 N 2 O 1
pa Chrg 1
462.84327
464.84032
460.84532
466.83828
463.84663
465.84368
461.84867
467.84163
468.83533
470.84204
471.84539
473.84964
Relative Abundance
m/z
m/z

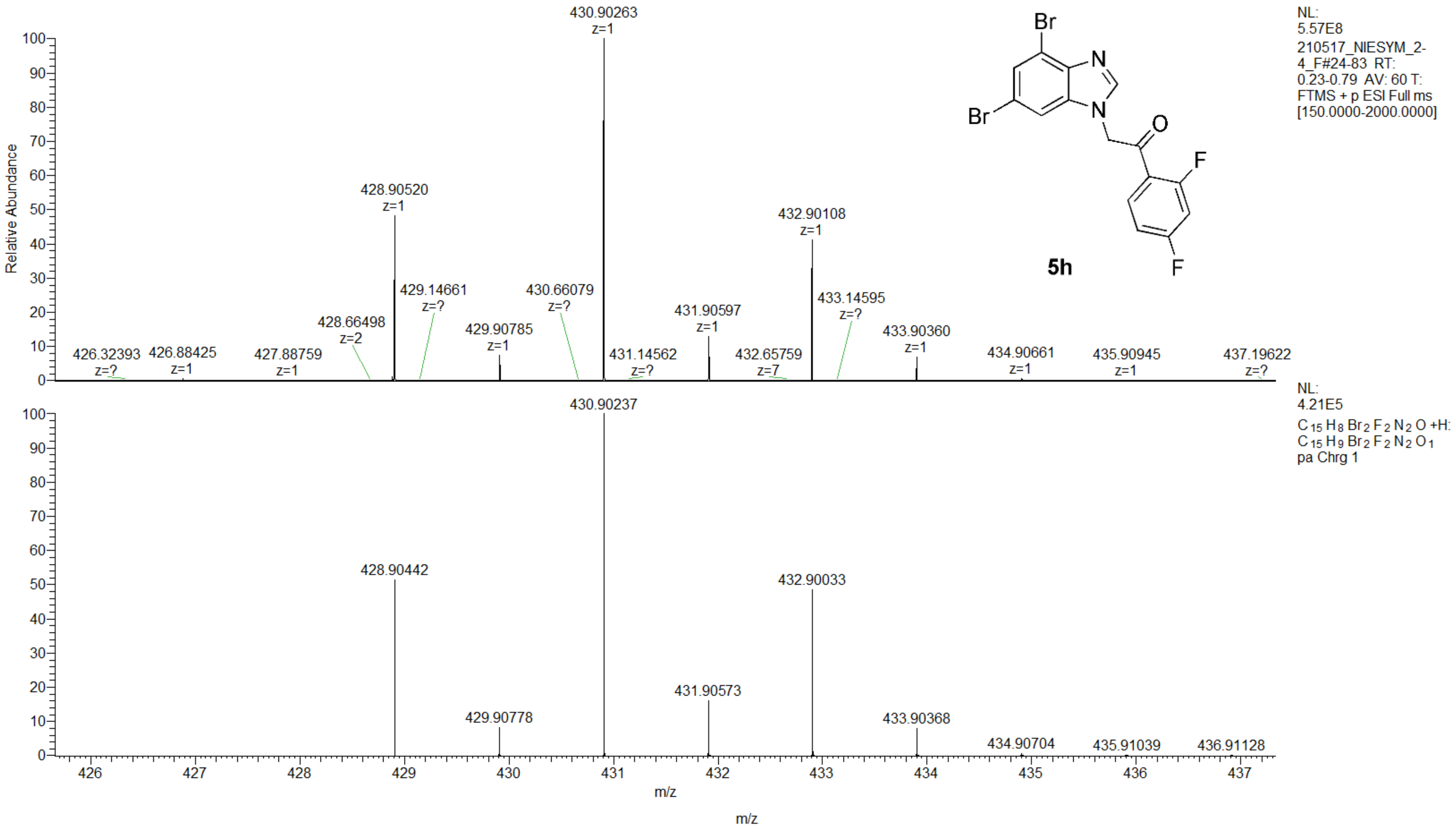

5h
NL: 5.57E8
210517_NIESYM_2-4_F#24-83 RT: 0.23-0.79 AV: 60 T: FTMS + p ESI Full ms [150.0000-2000.0000]
NL: 4.21E5
C 15 H 8 Br 2 F 2 N 2 O +H: C 15 H 9 Br 2 F 2 N 2 O 1 pa Chrg 1
Relative Abundance
m/z
430.90263 z=1
428.90520 z=1
432.90108 z=1
429.14661 z=?
430.66079 z=?
431.90597 z=1
433.14595 z=?
428.66498 z=2
429.90785 z=1
433.90360 z=1
426.32393 z=?
426.88425 z=1
427.88759 z=1
431.14562 z=?
432.65759 z=7
434.90661 z=1
435.90945 z=1
437.19622 z=?
430.90237
428.90442
432.90033
431.90573
429.90778
433.90368
434.90704
435.91039
436.91128

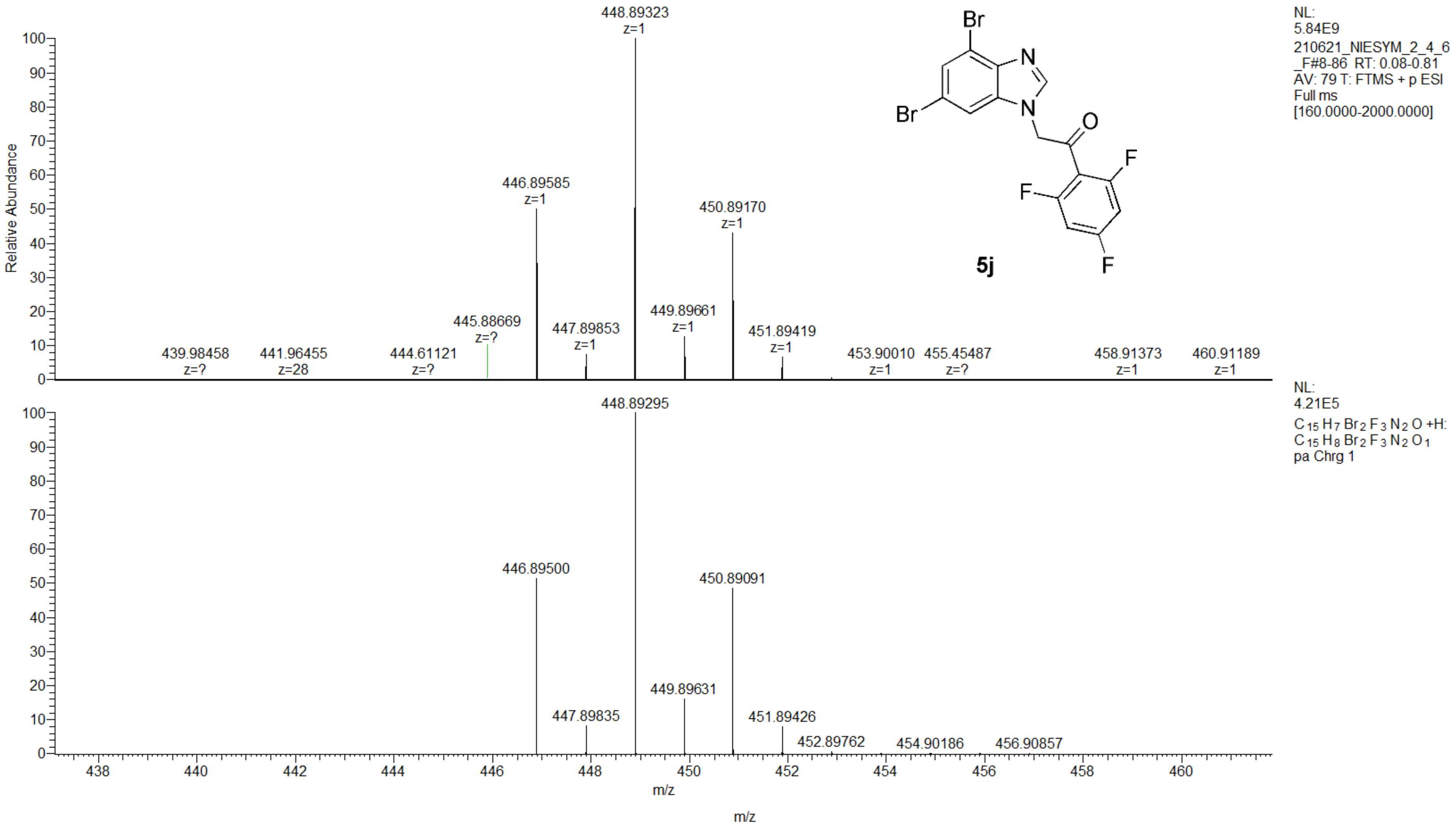
NL:
5.84E9
210621_NIESYM_2_4_6
_F#8-86 RT: 0.08-0.81
AV: 79 T: FTMS + p ESI
Full ms
[160.0000-2000.0000]
Relative Abundance
448.89323 z=1
446.89585 z=1
450.89170 z=1
449.89661 z=1
451.89419 z=1
447.89853 z=1
445.88669 z=?
439.98458 z=?
441.96455 z=28
444.61121 z=?
453.90010 z=1
455.45487 z=?
458.91373 z=1
460.91189 z=1
5j
NL:
4.21E5
C 15 H 7 Br 2 F 3 N 2 O +H:
C 15 H 8 Br 2 F 3 N 2 O 1
pa Chrg 1
448.89295
446.89500
450.89091
449.89631
447.89835
451.89426
452.89762
454.90186
456.90857
m/z
m/z

Table S1. Characteristics of 4,6-dibromidebenzimidazol *N*-phenacyl derivates

Comp.	Molecular formula	Structural formula
**5b**	$C_{15}H_9Br_2FN_2O$	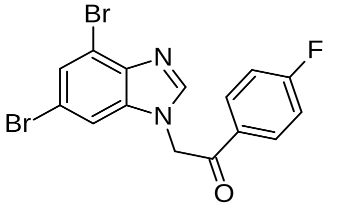
**5e**	$C_{15}H_8Br_2Cl_2N_2O$	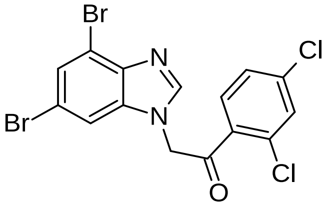
**5f**	$C_{15}H_8Br_2Cl_2N_2O$	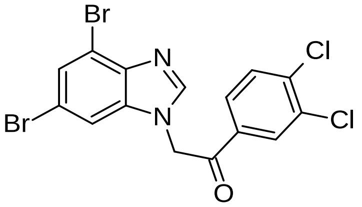
**5j**	$C_{15}H_7Br_2F_3N_2O$	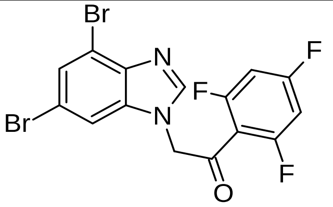
**5d**	$C_{15}H_9Br_3N_2O$	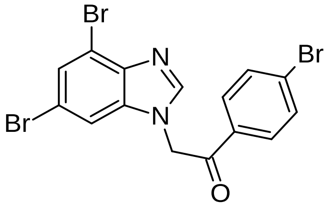
**5h**	$C_{15}H_8Br_2F_2N_2O$	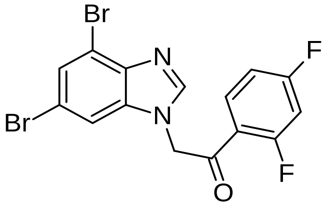

4. Antifungal assays

Figure S1. Cell growth inhibition under **4b**

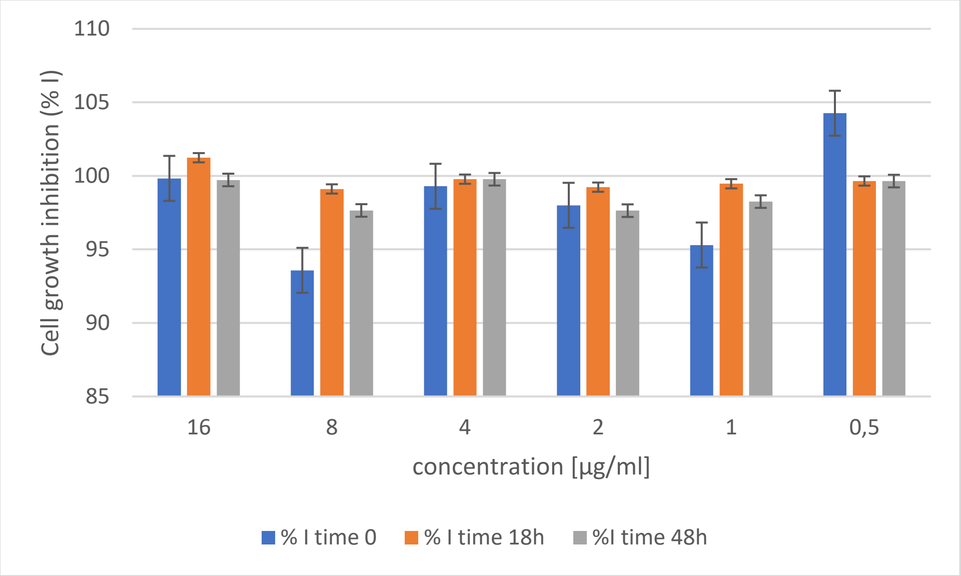

Legend: % I time 0 means cell growth inhibition under **4b** after time 0; % I 18 h means cell growth inhibition under **4b** after time 18h; % I 48 h means cell growth inhibition under 4b after time 48h.

Figure S2. Cell growth inhibition under **4c**

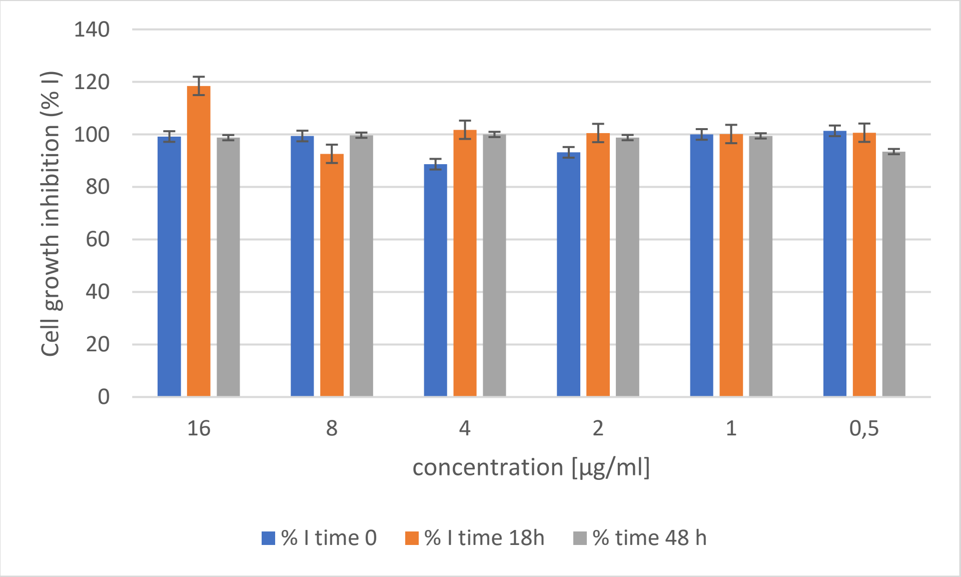

Legend: % I time 0 means cell growth inhibition under **4c** after time 0; % I 18 h means cell growth inhibition under **4c** after time 18h; % I 48 h means cell growth inhibition under **4c** after time 48h.

Figure S3. Cell growth inhibition under **4d**

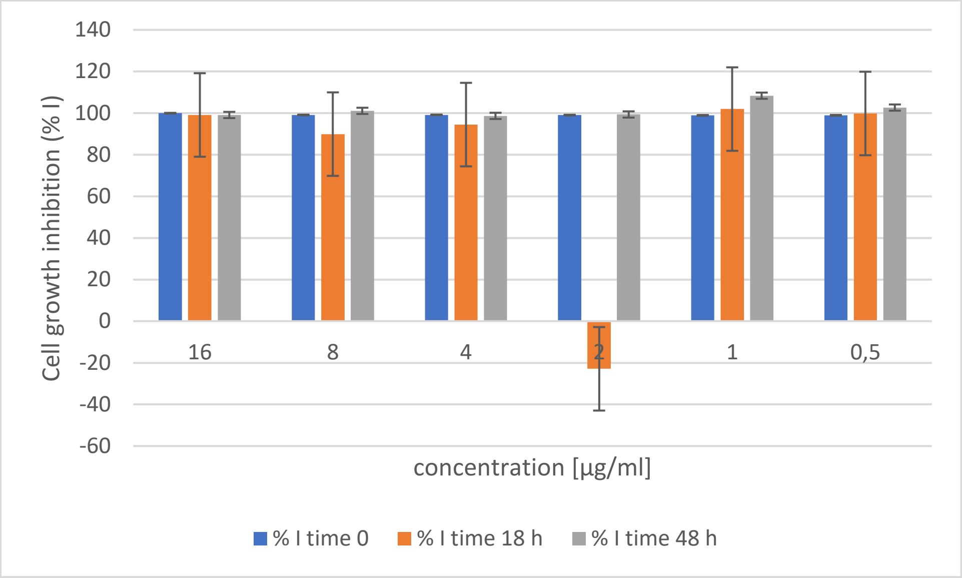

Legend: % I time 0 means cell growth inhibition under **4d** after time 0; % I 18 h means cell growth inhibition under **4d** after time 18h; % I 48 h means cell growth inhibition under **4d** after time 48h.

Figure S4. Cell growth inhibition under **4e**

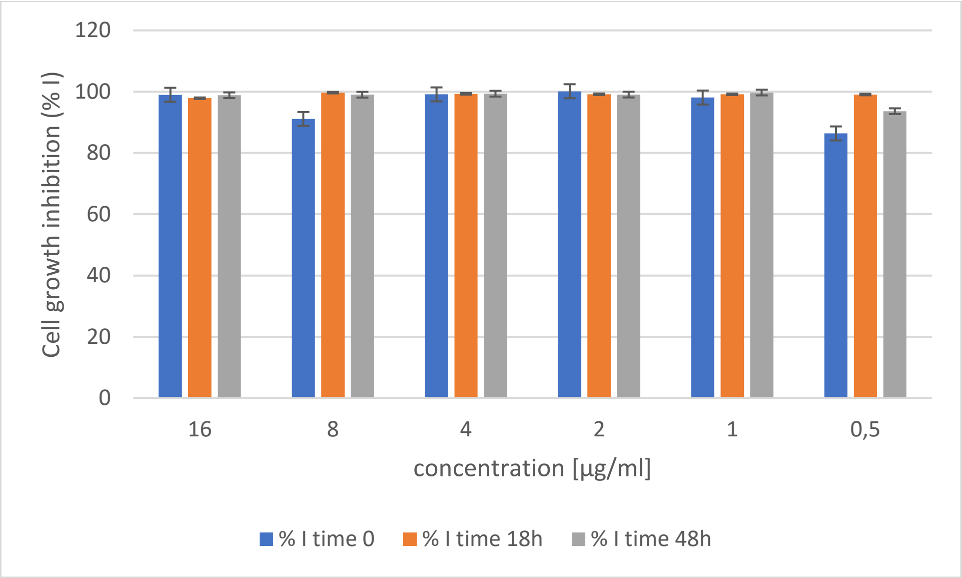

Legend: % I time 0 means cell growth inhibition under **4e** after time 0; % I 18 h means cell growth inhibition under **4e** after time 18h; % I 48 h means cell growth inhibition under **4e** after time 48h.

Figure S5. Cell growth inhibition under **4f**

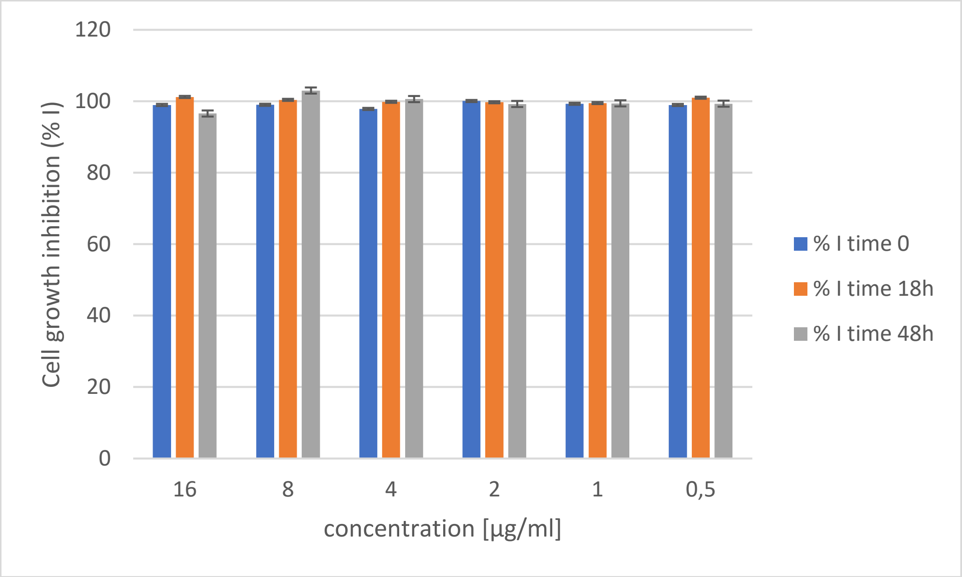

Legend: % I time 0 means cell growth inhibition under **4f** after time 0; % I 18 h means cell growth inhibition under **4f** after time 18h; % I 48 h means cell growth inhibition under **4f** after time 48h.

Figure S6. Cell growth inhibition under **4g**

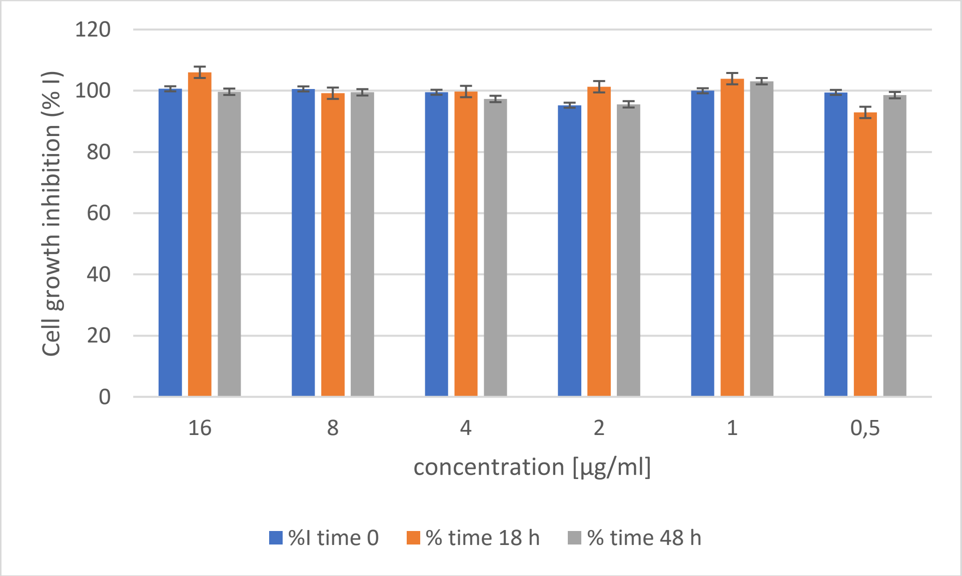

Legend: % I time 0 means cell growth inhibition under **4g** after time 0; % I 18 h means cell growth inhibition under **4g** after time 18h; % I 48 h means cell growth inhibition under **4g** after time 48h.

Figure S7. Cell growth inhibition under **4h**

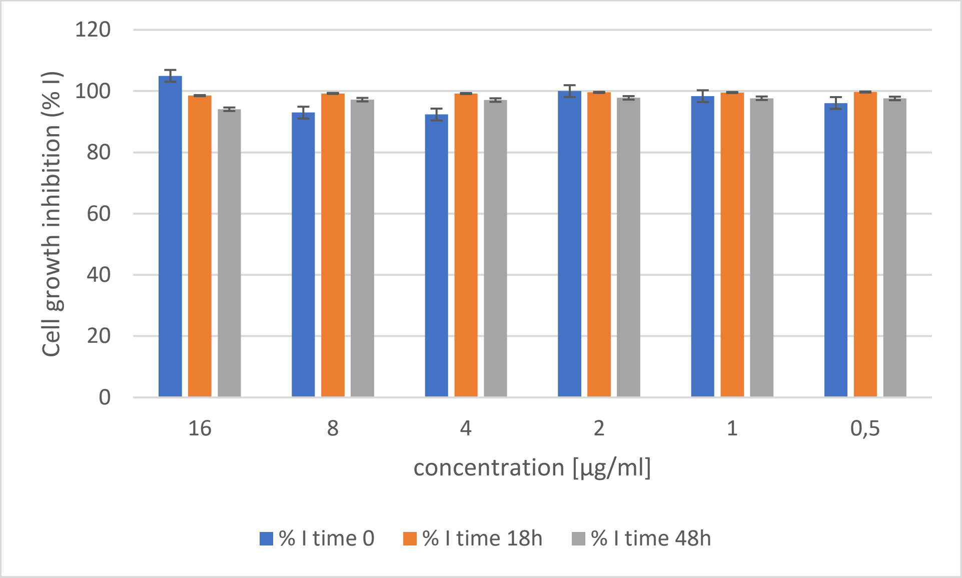

Legend: % I time 0 means cell growth inhibition under **4h** after time 0; % I 18 h means cell growth inhibition under **4h** after time 18h; % I 48 h means cell growth inhibition under **4h** after time 48h.

Figure S8. Cell growth inhibition under **4i**

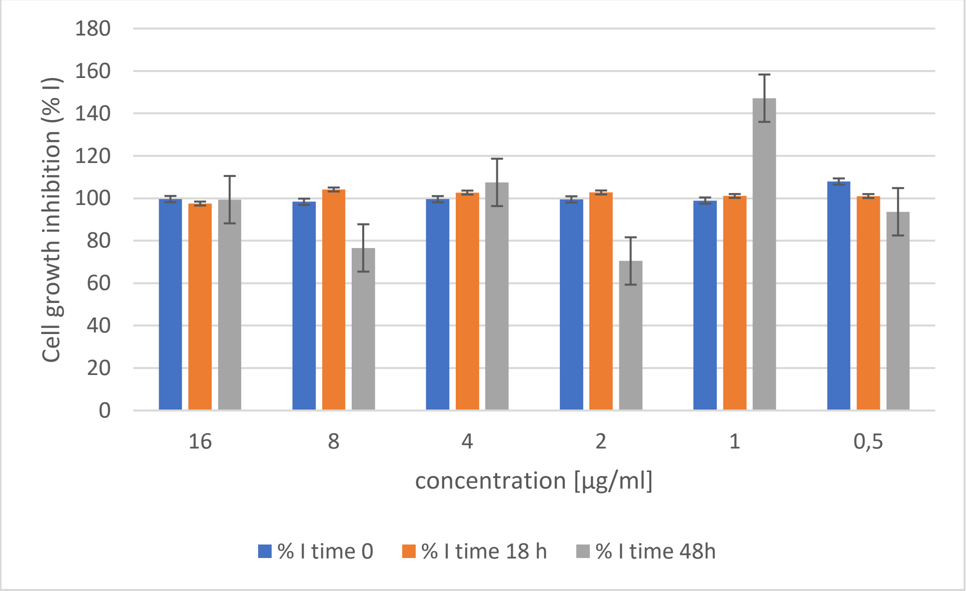

Legend: % I time 0 means cell growth inhibition under **4i** after time 0; % I 18 h means cell growth inhibition under **4i** after time 18h; % I 48 h means cell growth inhibition under **4i** after time 48h.

Figure S9. Cell growth inhibition under **5a**

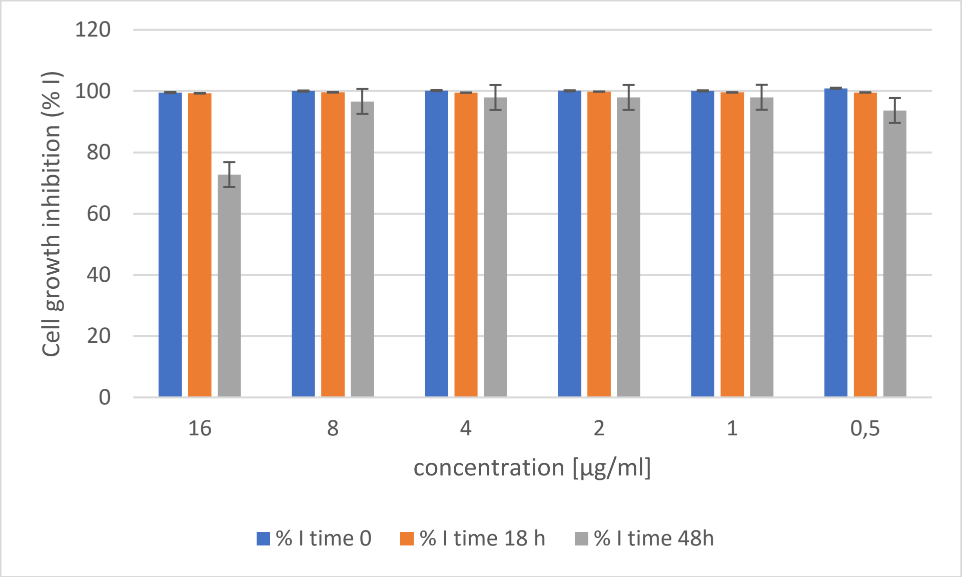

Legend: % I time 0 means cell growth inhibition under **5a** after time 0; % I 18 h means cell growth inhibition under **5a** after time 18h; % I 48 h means cell growth inhibition under **5a** after time 48h.

Figure S10. Cell growth inhibition under **5c**

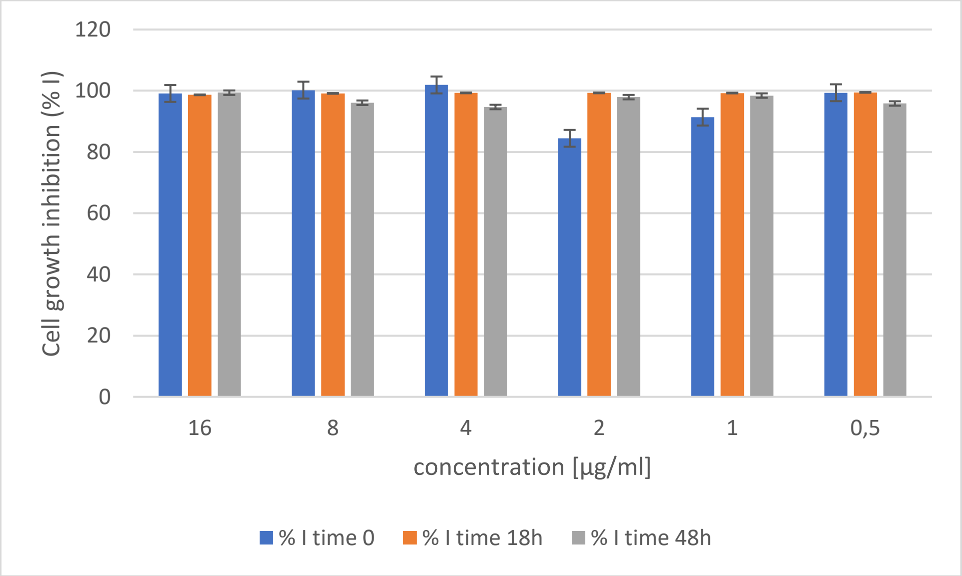

Legend: % I time 0 means cell growth inhibition under **5c** after time 0; % I 18 h means cell growth inhibition under **5c** after time 18h; % I 48 h means cell growth inhibition under **5c** after time 48h.

Figure S11. Cell growth inhibition under **5g**

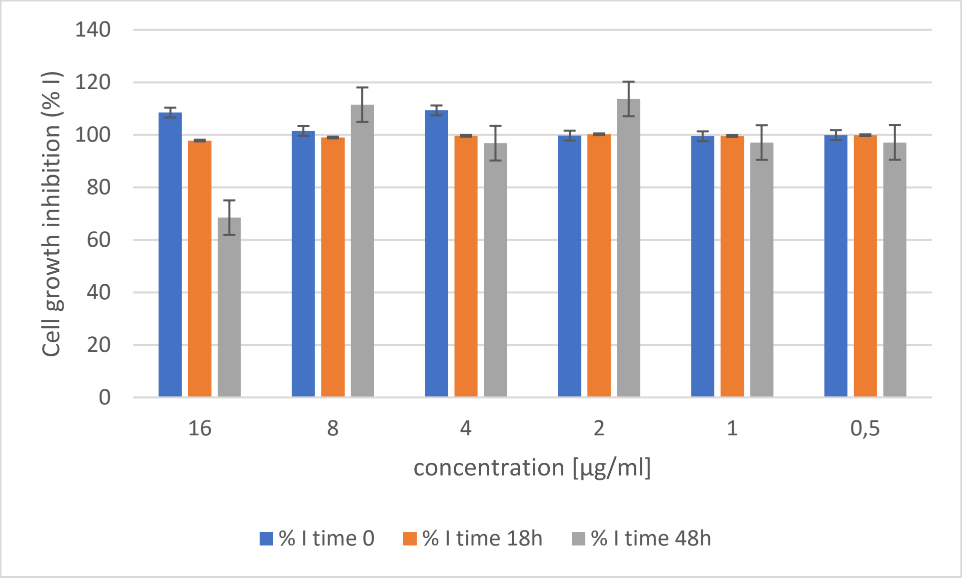

Legend: % I time 0 means cell growth inhibition under **5g** after time 0; % I 18 h means cell growth inhibition under **5g** after time 18h; % I 48 h means cell growth inhibition under **5g** after time 48h.

Figure S12. Cell growth inhibition under **5i**

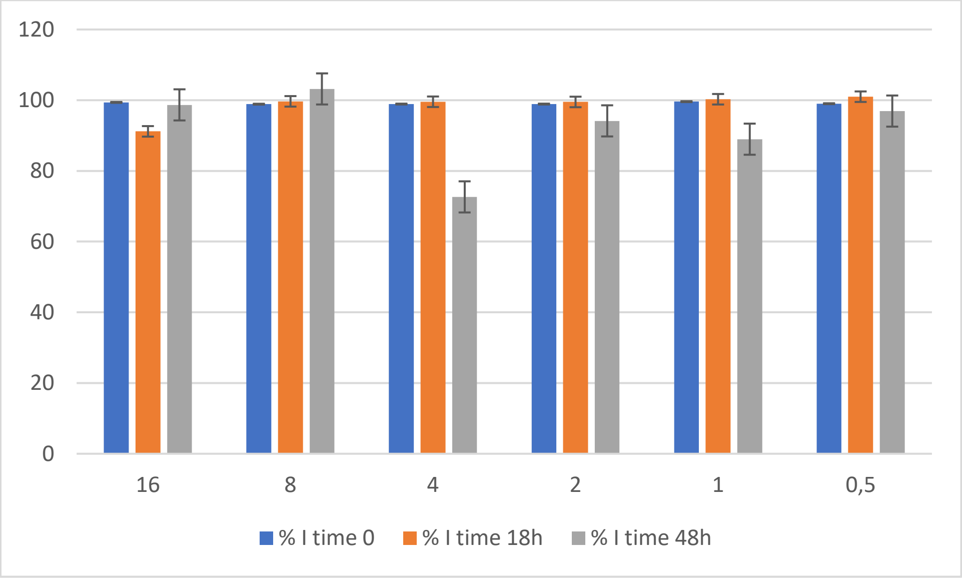

Legend: % I time 0 means cell growth inhibition under **5i** after time 0; % I 18 h means cell growth inhibition under **5i** after time 18h; % I 48 h means cell growth inhibition under **5i** after time 48h.

Table S2. Viability of Vero cells treated with phenacyl dibromide derivatives

**Viability of Vero cells [%]**			
Conc [µg/ml]	Comp.		
	**5h**	**5e**	**5f**
256	4.7±8	23.4±12	20.0±10
128	22.0±8	57.4±8	10.6±11

64	29.1±8	10.4±18	7.4±8
32	104.3±7	91.2±5	46.5±5
16	105.5±6	98.2±3	104.0±6
8	103.2±9	101.7±5	106.3±3
4	102.3±10	99.0±4	107.0±3
2	106.3±9	92.9±4	105.8±2
1	104.6±6	96.9±3	103.2±2
0.5	102.7±5	100.0±10	104.9±5
0.25	104.0±5	98.2±4	104.2±5
0.125	104.2±5	96.1±7	111.6±4

Legend: data is presented as [mean±SD]

Table S3. Cytotoxicity of phenacyl dibromide derivatives

**Cytotoxicity [%]**			
Conc [µg/ml]	Comp.		
	**5h**	**5e**	**5f**
256	77.3±8	58.4±12	62.3±10
128	60.0±8	24.3±8	71.7±11
64	53.0±8	71.4±18	75.0±8
32	0±7	0±5	35.8±5
16	0±6	0±3	0±6
8	0±9	0±5	0±3
4	0±10	0±4	0±3
2	0±9	0±4	0±2
1	0±6	0±3	0±2
0.5	0±5	0±10	0±5
0.25	0±5	0±4	0±5
0.125	0±5	0±7	0±4

Legend: data is presented as [mean±SD]

Figure S13. Cytotoxicity of phenacyl dibromide derivatives

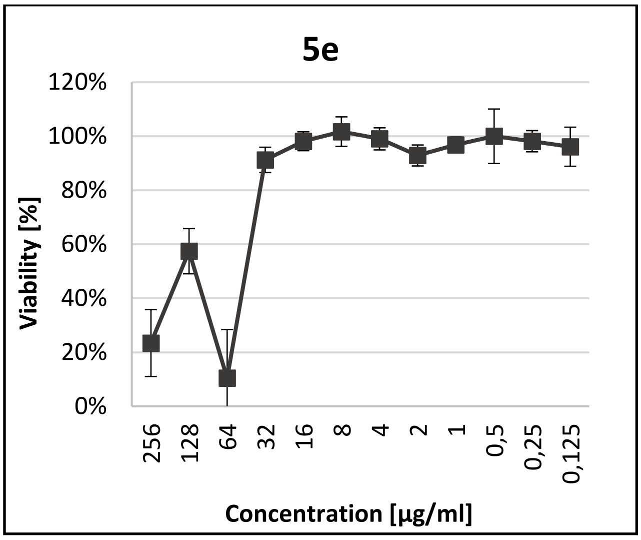

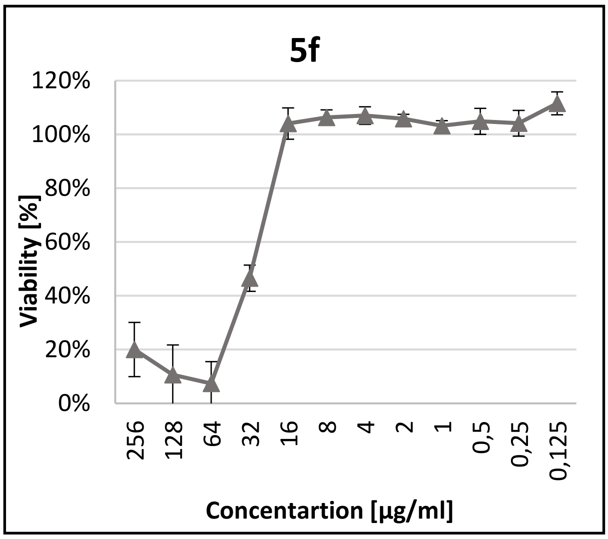

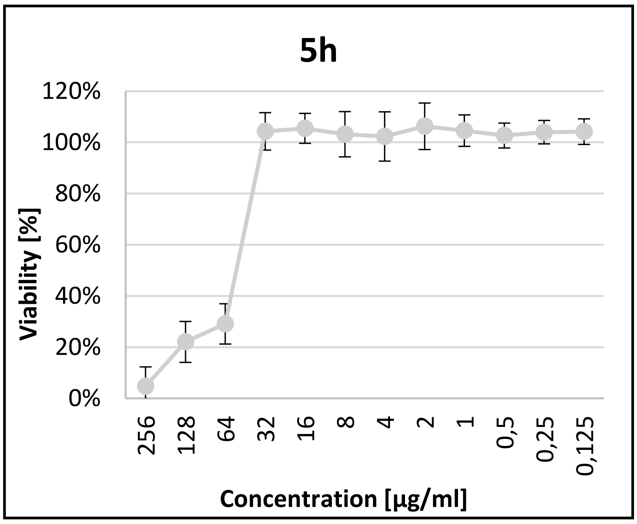

Table S4. Change in ROS content [ΔC±RSD] in post growth medium of *C. albicans* SC5314, *C. albicans* SPZ176 or *C. neoformans* SPZ173 treated with **5h** compared to untreated control

**Conc. [µg/ml]**	**Fungal strain**		
	***C. albicans* SC5314**	***C. albicans* SPZ176**	***C. neoformans* SPZ173**
**160**	-61±1	-45±1	84±1
**16**	-5±1	-78±1	143±2
**4**	-9±1	22±15	198±2
**0**	0±1	0±0	0±2

Table S5. Fractions of live, necrotic, early, and late apoptosis *C. albicans* SPZ176 and *C. neoformans* SPZ173 cells and protoplasts (P), treated with different concentration of **5h**.

	Concentration [µg/ml]	Fraction of cell population [%]			
		Live	Necrotic	Early apoptosis	Late apoptosis
C. albicans	160	20.53	78.39	0.21	0
	16	97.48	2.52	0	0
	4	97.53	2.47	0	0
	0	96.24	3.76	0	0
C. albicans	160	45.40	53.47	0.31	0.83
	16	89.02	10.98	0	0
	4	96.97	3.03	0	0
	0	98.23	1.77	0	0
C. neoformans	160	66.90	33.04	0.04	0.02
	16	82.01	17.97	0	0.02
	4	89.22	10.78	0	0
	0	93.91	6.19	0	0
C. neoformans	160	63.65	36.32	0	0.03
	16	74.51	25.47	0	0.02
	4	89.15	10.85	0	0
	0	92.49	7.49	0.01	0
